# Flp, a Fis‐like protein, contributes to the regulation of type III secretion and virulence processes in the phytopathogen *Xanthomonas campestris* pv. *campestris*

**DOI:** 10.1111/mpp.12818

**Published:** 2019-05-14

**Authors:** Ming Leng, Zhuo‐Jian Lu, Zuo‐Shu Qin, Yan‐Hua Qi, Guang‐Tao Lu, Ji‐Liang Tang

**Affiliations:** ^1^ State Key Laboratory for Conservation and Utilization of Subtropical Agro‐bioresources, College of Life Science and Technology Guangxi University 100 Daxue Road Nanning Guangxi 530004 China

**Keywords:** Fis‐like protein, *hrpX* promoter, type III secretion system, virulence, *Xanthomonas*

## Abstract

The ability of the plant pathogen *Xanthomonas campestris* pv. *campestris* (*Xcc*) to cause disease is dependent on its ability to adapt quickly to the host environment during infection. Like most bacterial pathogens, *Xcc* has evolved complex regulatory networks that ensure expression and regulation of their virulence genes. Here, we describe the identification and characterization of a Fis‐like protein (named Flp), which plays an important role in virulence and type III secretion system (T3SS) gene expression in *Xcc*. Deletion of *flp* caused reduced virulence and hypersensitive response (HR) induction of *Xcc* and alterations in stress tolerance. Global transcriptome analyses revealed the Flp had a broad regulatory role and that most T3SS HR and pathogenicity (*hrp*) genes were down‐regulated in the *flp* mutant. *β*‐glucuronidase activity assays implied that Flp regulates the expression of *hrp* genes via controlling the expression of *hrpX*. More assays confirmed that Flp binds to the promoter of *hrpX* and affected the transcription of *hrpX* directly. Interestingly, the constitutive expression of *hrpX* in the *flp* mutant restored the HR phenotype but not full virulence. Taken together, the findings describe the unrecognized regulatory role of Flp protein that controls *hrp* gene expression and pathogenesis in *Xcc*.

## Introduction

Xanthomonads are Gram‐negative bacteria that are known to cause disease in a range of important crops worldwide. *Xanthomonas campestris* pv. *campestris* (*Xcc*) is one of the best studied of these as it is the causative agent of black rot disease in crucifers, which include many important vegetable brassica crops (Swings and Civerolo, 1993; Vicente and Holub, 2013). *Xcc* is also an important model for the study of microbe–plant interactions because of its genetic tractability and cultivability. For these reasons, the study of *Xcc* has provided a lot of insight into how plant pathogens can adapt to the host environment and cause disease. Studies have revealed many mechanisms that are important for disease and environmental adaptation, including extracellular enzymes (protease, mannanase, etc.), extracellular polysaccharides (EPS), diffusible signal factor (DSF)‐dependent cell–cell signalling, and proteins secreted by the type II secretion system (T2SS), type III secretion system (T3SS), type IV secretion system (T4SS) and more recently type VI secretion system (T6SS) (He and Zhang, 2008; Büttner and Bonas, 2010; Ryan *et al.*, 2011, 2015; Notti and Stebbins, 2016; Zhou *et al.*, 2017). In *Xcc*, one of the best‐studied mechanisms that contributes to virulence is the T3SS apparatus. This complex system is encoded by over 20 hypersensitive response and pathogenicity (*hrp*) genes that when silenced lead to the loss of *Xcc*'s ability to cause full virulence and hypersensitive response (HR) induction (Alfano and Collmer, 1997; Lindgren, 1997). The activation of *hrp* genes, as well as some genes that encode secreted effector proteins, is controlled by two main regulators: HrpG (OmpR family regulator) and HrpX (AraC‐type transcriptional activator) (Huang *et al.*, 2009; Koebnik *et al.*, 2006; Wengelnik *et al.*, 1996; Wengelnik and Bonas, 1996).

Several other proteins have been shown to control the expression of *hrp* genes. HpaS, a sensor kinase that putatively constitutes a two‐component signal transduction system with HrpG, positively regulates the expression of *hrp* genes (Li *et al.*, 2014). The zinc uptake regulator (Zur), a key regulator of zinc homeostasis belonging to the Fur family of transcription factors, positively regulates the *hrp* gene expression via HrpX (Huang *et al.*, 2009). HpaR1 (*hrp*‐associated regulator), a global regulator belonging to the YtrA subfamily of the GntR family, appears to indirectly regulate the expression of *hrp* genes via HrpG (An *et al.*, 2011). In addition, HpaP, a novel regulatory protein with ATPase and phosphatase activity, regulates the expression of *hrp* genes by controlling the expression of *hrpX*. However, this is unlikely to be by a direct action (Cui *et al.*, 2018).

Despite these advances, considerable work is still required to understand the regulatory mechanisms associated with control of T3SS and virulence in this important bacterial plant pathogen. In the present study, we report the identification and characterization of a Fis‐like protein (Flp), a previously unreported protein regulator of *hrp* and other virulence determinants in *Xcc*. We demonstrate that this protein contributes to virulence, HR induction and a series of other cellular functions in *Xcc*. We present evidences that Flp is likely to regulate the expression of *hrp* genes by controlling the expression of *hrpX* via directly binding to its promoter.

## Results

### Flp is important for pathogenicity in *Xcc*

In our earlier work aimed at establishing a general view of the factors that contribute to pathogenicity in *Xcc*, we screened a series of mutants which were constructed using a suicide vector (pK18*mob*) strategy (Windgassen *et al.*, 2000). Virulence assays using the host plant cabbage (*Brassica oleracea*) showed that one of these mutants, 0520nk (Table S1), caused weaker symptoms of disease when compared with the wild‐type *Xcc* strain. The 0520nk mutant had a disrupted gene which corresponded to open reading frame (ORF) *XC_0520* in the genome of *Xcc* strain 8004 (accession number CP000050). The *XC_0520* gene encodes a putative DNA‐binding protein, which on further analysis with the SMART (Simple Modular Architecture Research Tool) program (http://smart.embl-heidelberg.de) has 40% identity with the Fis (factor for inversion stimulation) protein that is found in many other bacteria, including *Pseudomonas aeruginosa*, *Escherichia coli* and *Yersinia pseudotuberculosis* (Fig. S1).

**Figure 1:**
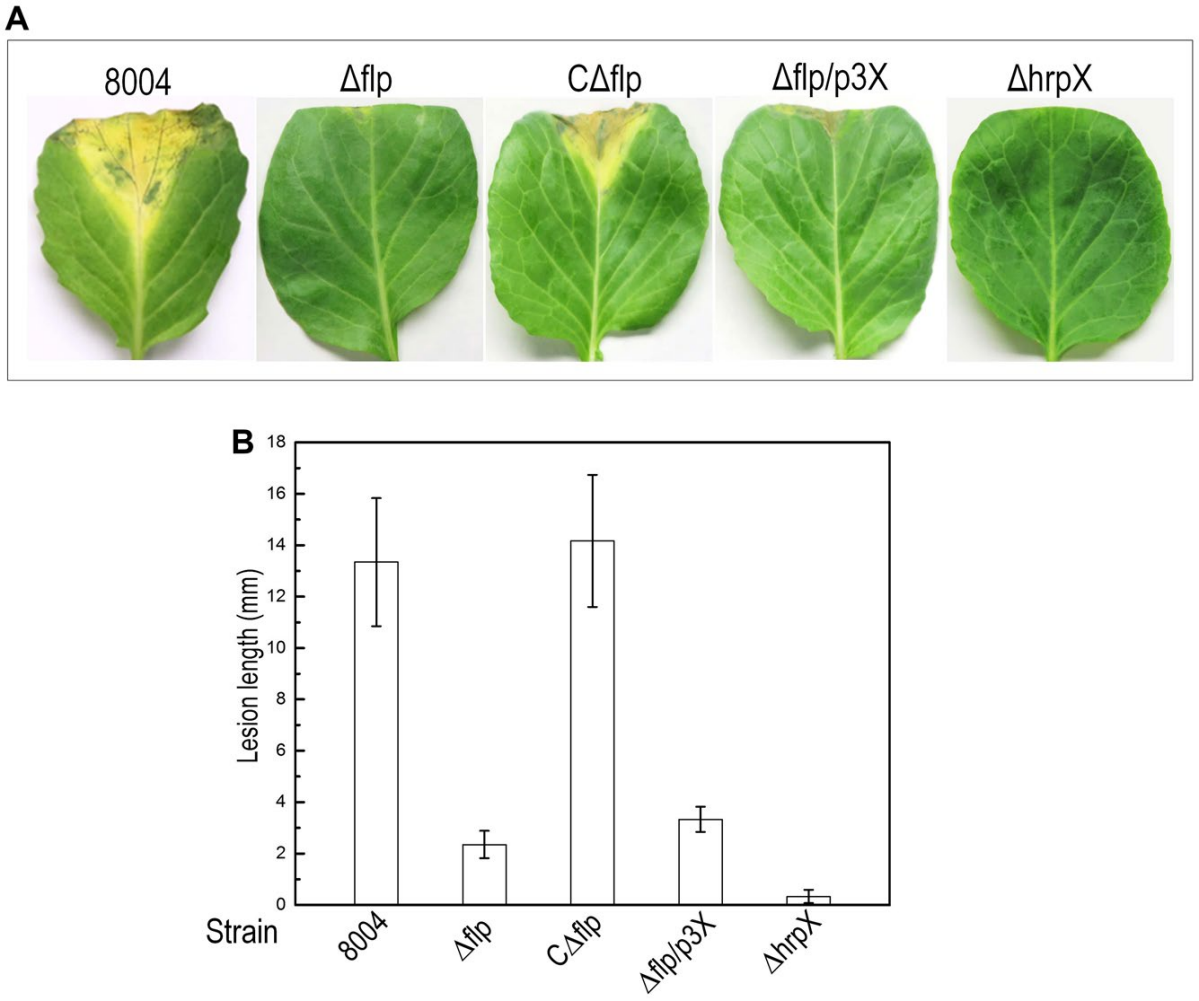
Flp plays a role in virulence in *Xanthomonas campestris* pv.* campestris (Xcc)*. The *Xcc* wild‐type strain 8004, *flp* deletion mutant Δflp, complemented strains CΔflp and Δflp/p3X (Δflp constitutively expressing *hrpX*), and *hrpX* deletion mutant ΔhrpX (negative control) were cultured in NYG medium overnight and then adjusted to 1 × 10^6^ CFU/mL in sterile distilled water. 30‐day‐old cabbage (*Brassica oleracea* 'Jingfeng No.1') was inoculated with bacterial suspensions of different *Xcc* strains by the leaf‐clipping method. (A) Infected cabbage leaves with *Xcc* strains showing symptoms as lesions 10 days post‐inoculation. (B) Lesion lengths were scored 10 days post‐inoculation. Values for means and standard deviation (SD) from 30 inoculated leaves in one experiment are indicated. The experiment was repeated three times.

In order to explore the detailed function of XC_0520 (herein Flp [Fis‐like protein]) in *Xcc*, we constructed a clean deletion removing the *flp* gene and designating the strain Δflp (see experimental procedures). Simultaneously, a complemented strain was constructed by introducing the recombinant plasmid, which carried the *flp* coding sequence, into the mutant Δflp. The resulting complemented strain was named CΔflp (Table S1). To confirm that deletion of *flp* influences the virulence in *Xcc*, the newly constructed strains were tested by inoculating into cabbage (*B. oleracea*) using leaf clippings (see Experimental procedures). Consistent with our previous findings, Δflp induced a significantly shorter lesion length compared with wild‐type (*P* < 0.05 by Student's *t*‐test) (Fig. 1). Importantly, the complementary strain CΔflp showed similar virulence symptoms (lesion length) to wild‐type (Fig. 1).

### Flp influences the regulation of extracellular polysaccharide, motility, stress tolerance and extracellular enzymes production

To explore if Flp manipulated specific functions that are known to be required for pathogenesis in *Xcc*, we conducted a series of basic phenotypic tests to assess extracellular polysaccharide (EPS) production, extracellular enzymes (cellulase and amylase), cell motility and the adaption to stress and antimicrobials.

**Figure 2:**
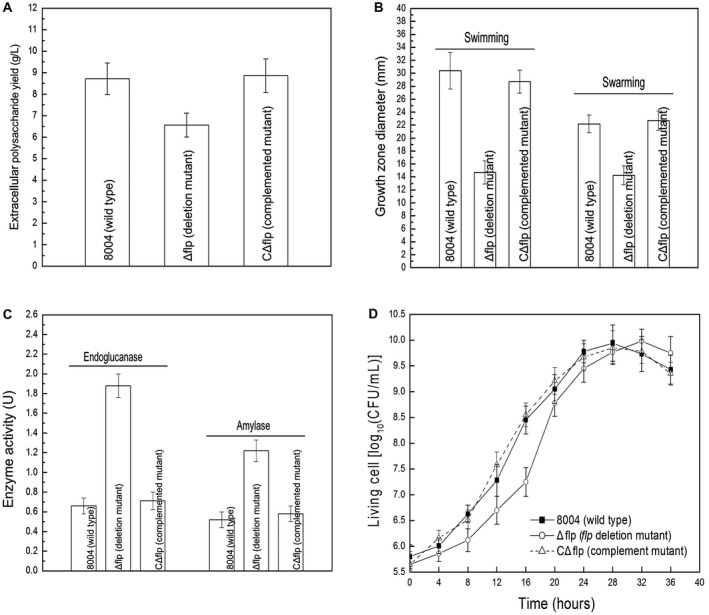
Flp positively regulates EPS production and motility, and negatively regulates the activities of extracellular enzymes in *Xanthomonas campestris* pv. *campestris* (*Xcc*). (A) The *flp*‐mutant Δflp produced significantly less EPS (*P* < 0.05 by Student's *t*‐test) compared to the wild‐type strain. Mean weight of EPS extracted from the Δflp mutant, the wild‐type strain and the CΔflp complemented strain. (B) Mean measurements of colony diameters of *Xcc* strains on ‘swim’ (0.28% agar) medium and ‘swarm’ (0.6% agar) medium after 3 and 4 days' incubation at 28 °C, respectively. Data are shown as mean ± SD (standard deviation). (C) Mean relative quantity of extracellular endoglucanase (cellulase) and amylase in *Xcc* strains inoculated into 100 mL NY medium. (D) Growth of *Xcc* strains in complex media. The strains were inoculated into NYG medium with the same final density of 0.01 (OD_600_). Growth of the strains was recorded at intervals of 4 h.

The results showed that the *flp*‐mutant strain Δflp displayed decreased EPS production (Fig. 2A) and motility (swimming and swarming, tested on 0.28% w/v agar plates and 0.6% w/v agar plates, respectively) (Fig. 2B). However, EPS production and motility of the CΔflp complemented strain were similar to the wild‐type under the conditions tested (Fig. 2A,B).

When extracellular enzymes in the *Xcc* wild‐type strain, Δflp mutant strain and CΔflp complemented strain were compared, positive differences were seen. The results show that the Δflp mutant exhibits a significant enhancement in cellulase and amylase secretion (*P* < 0.05 by Student's *t*‐test) (Fig. 2C). Moreover, enhancement in the activity of extracellular enzymes could be restored to wild‐type levels in the CΔflp complemented strain (Fig. 2C).

The growth characteristics of the *Xcc* strains in liquid medium nutrient‐yeast‐glycerol (NYG) were also investigated. Results revealed that the *flp*‐mutant Δflp displayed small changes in growth properties. The mutant had a reduced growth rate compared to that of the wild‐type strain in the early exponential phase (Fig. 2D). However, the growth rate was recovered in the mid‐exponential phase and the Δflp mutant grew with a rate exceeded that of the wild‐type in the late exponential phase.

**Figure 3:**
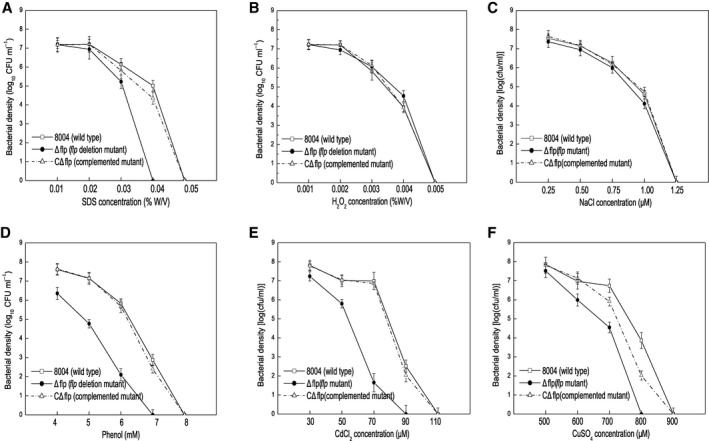
Flp is required for stress tolerance in *Xanthomonas campestris* pv. *campestris* (*Xcc*). Survival experiments performed by subculturing strains overnight on fresh NYG agar plates supplemented with different concentrations of SDS (A), oxidant H_2_O_2 _(B), hyperosmosis NaCl (C), phenol (D) and heavy metal salts CdCl_2_ (E) and CuSO_4_ (F). The surviving bacterial colonies on the plates were counted after incubation for 3 days.

Additionally, differences were seen when the wild‐type strain, the Δflp mutant and the CΔflp complemented strain were assessed for their ability to adapt to environmental stresses (Fig. 3). For these experiments, kill curve assays were used in which the quantity of living cells on agar plates were supplied with various different concentrations of environmental stresses, including sodium dodecyl sulphate (SDS), H_2_O_2_, NaCl, phenol and heavy metal salt CdCl_2_ and CuSO_4_ (see Experimental procedures). These tests demonstrated that compared to the wild‐type, the Δflp mutant showed reduced survival in the presence of phenol, CdCl_2_, CuSO_4_ and SDS but not H_2_O_2_ and NaCl (Fig. 3). Importantly, in all cases the complementation strain responded similarly to the wild‐type (Fig. 3).

Taken together, these findings suggest that Flp regulates positively the EPS production, motility and some stress tolerance but appears to negatively regulate extracellular enzymes in *Xcc* under the conditions tested. Despite these observations, the mechanism of regulation by Flp in these cases remains enigmatic.

### Flp has an influence over the expression of genes involved in virulence and various adaptation processes in *Xcc*

**Figure 4:**
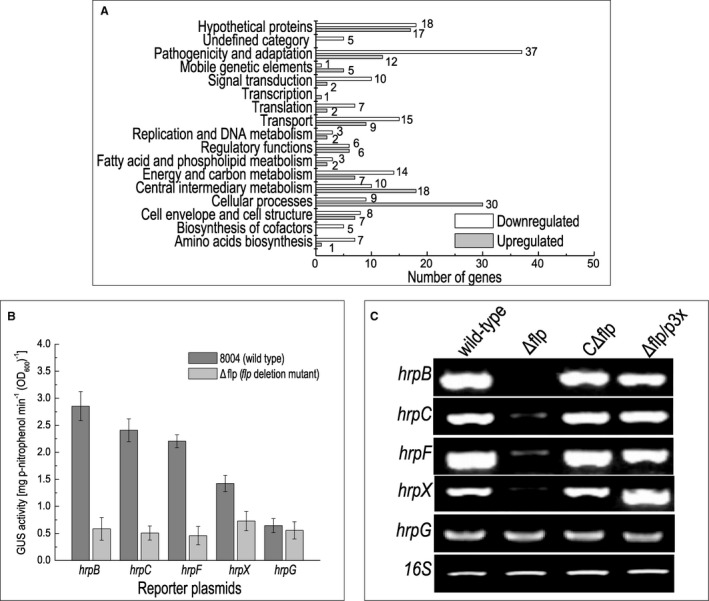
Flp is a global regulatory protein that affects the expression of a number of genes, including T3SS genes. (A) Functional categories of differential expressed genes in Δflp mutant. Genome‐scale transcriptome profiling of *Xanthomonas campestris* pv. *campestris* (*Xcc*) strains cultured in XVM2 were investigated by RNA sequencing, and 279 genes were found differentially expressed by two‐fold or more in Δflp mutant (Table S2). These genes were broadly categorized according to their biological function (He *et al*., 2007). Notably, due to cross‐talk between different metabolism pathways, some genes might be counted into different categories simultaneously. (B) ß‐glucuronidase (GUS) activity of *hrp* gene promoter‐*gusA* reporters in Δflp mutation and wild‐type backgrounds. Data shown are mean and standard deviation of triplicate measurements. The experiment was repeated twice, and similar results were obtained. (C) The expression levels of *hrp* genes in *Xcc* wild‐type strain 8004, Δflp, CΔflp and Δflp/p3X as measured by semi‐quantitative RT‐PCR.

To gain a greater understanding of the regulatory role of Flp in *Xcc* a set of global gene expression profiles was generated using RNA‐Seq. For this, selected *Xcc* strains were grown to the mid‐exponential phase (OD_600_ = 0.6) in XVM2, a medium that mimics more closely the nutrition environment of the plant (Astua‐Monge *et al.*, 2005). Following bacterial RNA extraction, library construction and sequencing, differential gene expression analysis was conducted on the generated data (see Experimental procedures). Of the 4273 annotated genes from the genome of *Xcc* strain 8004, 279 genes presented differentially expressed (fold changes ≥ 2.0), with 121 genes up‐regulated and 158 genes down‐regulated according to the transcriptome data (Fig. 4A, Table S2). In order to verify the transcriptome data, 16 genes that showed changes were selected randomly and reverse transcription‐polymerase chain reaction (RT‐PCR) was performed to examine the relative expression levels of these genes. All selected genes showed expression changes that were comparable with the transcriptome data (Table 1).

**Table 1:** Confirmation of RNA‐Seq gene expression data by semi‐quantitative RT‐PCR.

ID	Gene	Description	Expression level	Semi RT‐PCR WT/Δflp
*XC_3001*	*hpa2*	Lysozyme‐related protein Hpa2	2.97↓	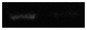
*XC_2324*		c‐di‐GMP phosphodiesterase A	4.33↓	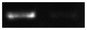
*XC_3657*	*copB*	Copper resistance protein B	2.58↓	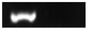
*XC_3129*	*pmrC*	Inner membrane protein	3.59↓	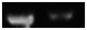
*XC_3597*	*hns*	DNA‐binding protein	3.03↓	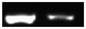
*XC_3437*	*lptD*	LPS‐assembly protein	2.29↓	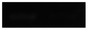
*XC_2004*	*avrXccC*	Avirulence protein	8.31↓	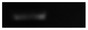
*XC_3694*	*ompW*	Outer membrane protein	2.59↓	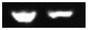
*XC_2827*	*hpaR*	MarR family transcriptional regulator	2.25↓	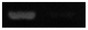
*XC_0158*		Xylosidase/arabinosidase	2.51↓	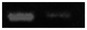
*XC_2659*	*gcd*	Quinoprotein glucose dehydrogenase	7.31↓	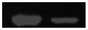
*XC_1273*	*trkA*	Voltage‐gated potassium channel	2.36↓	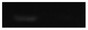
*XC_1314*	*lptC*	Lipopolysaccharide export system protein LptC	2.51↓	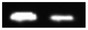
*XC_3652*	*fabB*	β‐ketoacyl‐[ACP] synthase	2.05↑	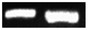
*XC_0783*	*celS*	Cellulase S	2.51↑	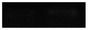
*16S rRNA*		Internal reference		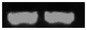

Note:

16 genes of the transcriptome data were chosen to validate the integral accuracy via semi‐quantitative RT‐PCR. RNA, extracted from the cultures of *Xanthomonas campestris* pv. *campestris* wild‐type (8004) and Δflp, respectively, was reverted into cDNA, and then cDNA was used as the template in semi‐quantitative PCR. In this study, false discovery rate (FDR) ≤ 0.05 and absolute value of log_2_ fold change ≥ 1 were used as the cut‐off values. The acquired results were accordant to the transcriptome data.↑, up‐regulated; ↓, down‐regulated.

Functional clustering analysis, according to the annotation of *Xcc* strain 8004 genome (Qian *et al.*, 2005), was carried out where the majority of the 279 genes regulated by Flp were assigned to the functional categories ‘pathogenicity and adaption’, ‘cellular process’, ‘central intermediary metabolism’, ‘transport’, ‘energy and carbon metabolism’, ‘cell envelope and cell structure’, ‘regulatory functions’ and ‘signal transduction’. The remaining genes were predicted to encode hypothetical proteins or have not been given a functional category to date (Fig. 4A; Table S2). The most dominant functional categories which genes were assigned to were ‘pathogenicity and adaption’ and ‘cellular processes’ (Fig. 4A). Notably, the deletion of Flp had an impact on genes belonging to the type III secretion system (T3SS). The expression of 16 *hrp* genes, *XC_3003* (*hrcC*), *XC_3004* (*hrcT*), *XC_3006* (*hrcN*), *XC_3007* (*hrpB5*), *XC_3009* (*hrcJ*), *XC_3010* (*hrpB2*), *XC_3012* (*hrcU*), *XC_3013* (*hrcV*), *XC_3015* (*hrcQ*), *XC_3016* (*hrcR*), *XC_3018* (*hpaA*), *XC_3019* (*hrpD5*), *XC_3022* (*hpaB*), *XC_3023* (*hrpW*), *XC_3025* (*hrpF*) and *XC_3076* (*hrpX*) was decreased in the Flp mutant compared to the wild‐type (Fig. 4A, Table S2).

Given that mutation of Flp leads to a significant reduction in virulence, it is feasible that the impact of Flp on the expression of pathogenicity related genes at the transcriptional level accounts for the phenotypes seen in the *flp* mutant.

### Flp regulates the expression of T3SS genes by altering the expression of key regulator HrpX

The gene transcription profile data presented suggest that Flp regulated the expression of the T3SS via *hrp* gene expression. To confirm this idea, we quantified the expression of several *hrp* operons (*hrpB*, *hrpC*, *hrpF*), and *hrpX* and *hrpG*, the key regulators of T3SS. This was achieved by using promoterless‐*gusA* transcriptional fusion reporters that we have deployed in previous studies (An *et al.*, 2011; Cui *et al.*, 2018; Huang *et al.*, 2009). Here we constructed a group of reporter plasmids for *hrpB*, *hrpC*, *hrpF*, *hrpX* and *hrpG* where the promoter sequence fused in front of the *gusA* gene so that the activity of *gusA* (Huang *et al.*, 2009). The reporter plasmids were introduced into the wild‐type and Δflp strains, respectively (Table S1). The reporter strains were cultured in XVM2 medium for 8 h, and the activities of *β*‐glucuronidase GUS were determined (see Experimental procedures). The results demonstrate that GUS activities for *hrpB*, *hrpC*, *hrpF* and *hrpX* reporters, but not the *hrpG* reporter, were significantly reduced in the Δflp deletion strain compared to the wild‐type (*P* < 0.05 by Student's *t*‐test) (Fig. 4B). A similar result was seen using an RT‐PCR assay, in which we compared RNA amounts of *hrpX* between wild‐type and Δflp cultured in media XVM2 (Fig. 4C). These observations indicate that Flp regulates the expression of *hrp* genes and T3SS, apparently by controlling the expression of *hrpX*.

### Flp enhances *hrpX* expression via binding to its promoter

**Figure 5:**
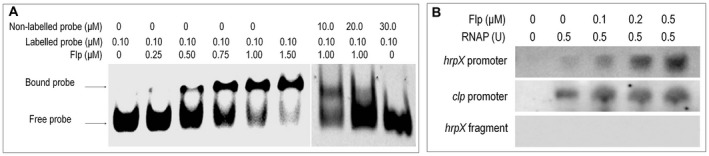
Flp regulates HrpX by interacting with the promoter sequence of *hrpX*. (A) Electrophoretic mobility shift assays (EMSA) to examine the interaction between the *hrpX* promoter sequence and Flp protein. In this experiment, DNA is acquired from purified PCR products of the *hrpX* promoter sequence with 5ʹ‐FAM labelled primers. The DNA is then incubated with purified Flp protein (protein final density 0, 0.25, 0.5, 0.75, 1 and 1.5 µM) and the *hrpX* promoter DNA is 0.10 µmol). A competition assay was also conducted with unlabelled DNA. When the concentration of unlabelled DNA increased from 10 to 30 µM, the Flp‐DNA complexity decreased, indicating the unlabelled DNA of *hrpX* brings competition to the 5'‐FAM‐labelled *hrpX* in forming DNA–protein complex with Flp. F, free DNA strips; R, retarded DNA strips. (B) *In vitro* transcription experiment using Flp protein (final density arranged from 0, 0.1, 0.20 and 0.5 µM) in the transcription system. A template DNA fragment containing the *clp* promoter (Liu *et al*., 2019) and a 121‐bp *hrpX* fragment extending from +1 to +121 relative to the TIS were used as controls.

The findings outlined above raise the question of how the Flp influences *hrpX* expression. One possible explanation is that Flp binds directly to the promoter of *hrpX*. To explore the potential interaction between Flp and *hrpX* promoter we conducted a set of electrophoretic mobility shift assays (EMSA) (see Experimental procedures). The 6 × His‐tagged expression construct of Flp was generated and the protein subsequently purified by nickel affinity column chromatography (see Experimental procedures). The purified 6 × His‐tagged Flp fusion protein caused a mobility shift of DNA probes spanning the promoter of *hrpX* [from +70 bp downstream to –131 bp upstream against the transcription initiation site (TIS) with a pair of 5ʹ FAM‐labelled primers]. The binding of the *hrpX* promoter appeared to increase with increasing concentrations of Flp protein (scale from 0 to 1500 nM) to the EMSA assays (Fig. 5A). Furthermore, the shifted bands also could be competed by excess of the unlabelled probes (Fig. 5A). Taken together, the data suggests that under the conditions used Flp binds to the upstream region of *hrpX*.

The fact that Flp binds to the promoter of *hrpX* suggests that Flp may directly regulate the expression of the *hrpX* gene. To validate this, we performed an *in vitro* transcription assay. Template DNA fragments of 311 bp, extending from –131 to +179 relative to the TIS of the *hrpX* promoter, were incubated with RNA polymerase holoenzyme from *Escherichia coli* with increasing amounts of purified 6 × His‐tagged Flp protein. The results show that, although a certain amount of *hrpX* transcripts could be generated without Flp protein, the *hrpX* transcription level was obviously increased when Flp protein was added to the reaction (Fig. 5B), suggesting that Flp could enhance *hrpX* transcription *in vitro*.

**Figure 6:**
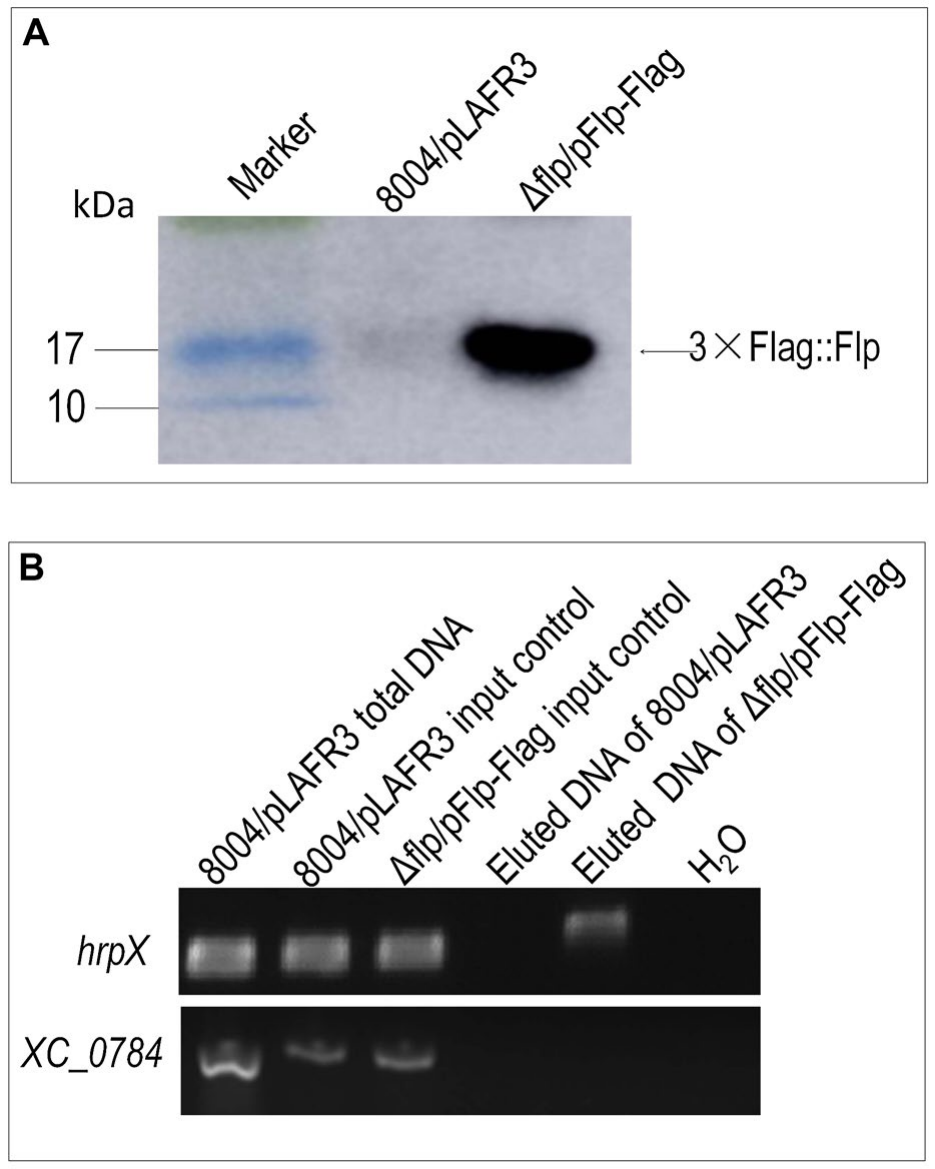
Chromatin immunoprecipitation (ChIP) assay showing that Flp binds to the *hrpX* promoter region *in vivo*. A *Xanthomonas campestris* pv. *campestris*  strain encoding Flp fused with the 3 × Flag peptide was created. The resulting Δflp/pFlp‐Flag strain was cultured in XVM2 medium for 12 h and ChIP samples were prepared. Anti‐Flag was added to the ChIP samples and incubated overnight. The bound DNA fragments and proteins were eluted. 8004/pLAFR3 was used as the control strain. (A) Western blotting of the eluted 3 × Flag::Flp fusion protein. Protein samples were separated by sodium dodecylsulphate‐polyacrylamide gel electrophoresis (SDS‐PAGE) and transferred to a polyvinylidene difluoride (PVDF) membrane. The presence of the fusion proteins was detected by anti‐Flag monoclonal antibody. (B) PCR detection of eluted DNA. DNA fragments containing the *hrpX* promoter were PCR amplified using the eluted DNA from the 3 × Flag::Flp protein as the template. Template DNA from a non‐conjugated ChIP sample was used as input control. Simultaneously, DNA fragments containing a cellulase S encoding gene *XC_0784* were amplified as positive and negative controls, respectively.

Whether or not Flp binds to the *hrpX* promoter *in vivo* was further estimated by chromatin immunoprecipitation (ChIP) assay. To do this, a wild‐type background strain expressing the Flp protein fused with 3 × Flag‐tag (3 × Flag::Flp) at the N‐terminus of Flp was first constructed (see Experimental procedures). *Xcc* strains were grown in XVM2 medium for 12 h and used for the ChIP assay. A western blot assay revealed that the 3 × Flag::Flp fusion protein could be eluted from the 3 × Flag::Flp expression strain Δflp/pFlp‐Flag, but not the control strain 8004/pLAFR3 (Fig. 6A). Using the eluted DNA from 3 × Flag::Flp protein as a template, a PCR product was obtained by the primer pair (Table S3) designed for amplification of the DNA fragment containing the *hrpX* promoter, but no product could be obtained by the primers (Table S3) for the promoter of the *XC_0784* gene (Fig. 6B), indicating that binding of Flp to *hrpX* promoter exists in *Xcc* cells.

### Flp regulates T3SS *in planta* by altering the expression of *hrpX*, which has an impact on the induction of HR and plant defence

**Figure 7:**
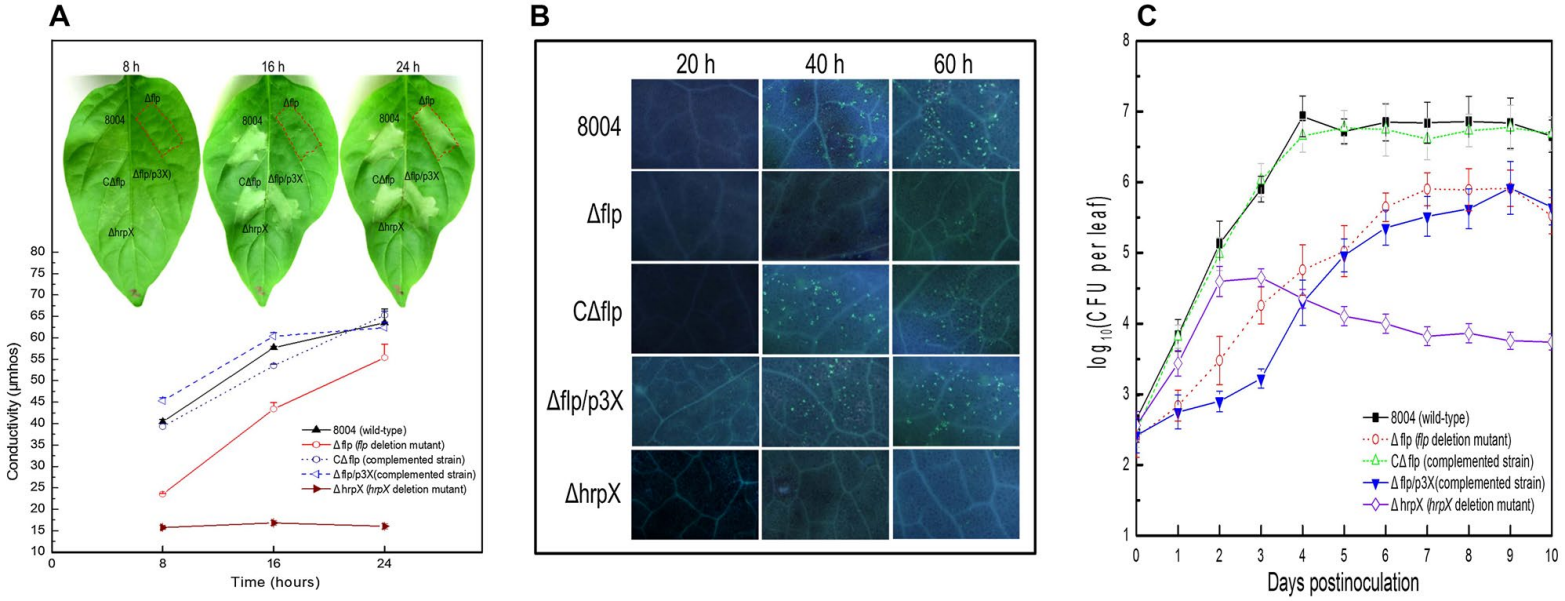
Flp is important for response to plant immunity and plant colonization. (A) Flp is required for HR induction in *Xanthomonas campestris* pv. *campestris* (*Xcc)*. HR symptoms observed after infiltration (upper part). The infiltration was performed on the fully expanded pepper (*Capsicum annuum* cv. ECW‐10R). The HR symptoms of strains *Xcc* wild‐type (8004), Δflp, CΔflp, Δflp/p3X and ΔhrpX were recorded at 8, 16 and 24 h post‐inoculation. There was electrolyte leakage from pepper leaves inoculated with *Xcc* strains (Down element). The conductivity of the infiltrating spots was measured by a DDS‐307A conductometer at 8, 16 and 24 h post‐inoculation, with four 0.4 cm^2^ leaf disks collected from the infiltrated area for each sample. Three samples were taken for each measurement in each experiment. Data are shown as mean and standard deviation. (B) Callose deposition within cabbage leaves inoculated with *Xcc* strains 8004, Δflp, CΔflp, Δflp/p3X and ΔhrpX using the infiltration method. Leaves were collected at intervals of 20 h and stained with aniline blue. The stained samples were observed under an Olympus BH‐2 epifluorescent microscope. Represented images are presented. (C) *In planta* growth curves of *Xcc* strains. 8004, Δflp, CΔflp, Δflp/p3X and ΔhrpX were inoculated onto cabbage leaves using leaf clipping methods. Four leaves were collected from every group of clipped leaves daily and homogenized in sterile water. The homogenates were diluted and plated on NYG plates. Bacterial CFU were counted after incubation for 3 days. Data are the means and standard deviations from three replicates.

Given that mutation of Flp leads to a reduction in the expression level of *hrp* genes, the influence of *flp* on the induction of HR was evaluated using an infiltration assay. Bacterial suspensions of the wild‐type, Δflp, CΔflp and a *hrpX* mutant (negative control) were inoculated into the leaves of non‐host pepper (*Capsicum annuum* cv. ECW‐10R) through the use of a pressurized syringe (see Experimental procedures). Following 8 h inoculation, the Δflp strain resembled the negative control by showing no visible HR symptoms (Fig. 7A). Conversely, the wild‐type and CΔflp strains induced comparable HR symptoms (Fig. 7A). It was not until after 16 h post‐inoculation that the Δflp strain appeared to induce any visual HR symptoms. These results suggest that the Δflp mutant had a delayed and weakened HR compared to the wild‐type.

To provide a quantitative assessment of HR induction an electrolyte leakage assay was used (see Experimental procedures). Here, leaf tissues within the infiltration areas were collected at three time points (8, 16 and 24 h) post‐inoculation of the *Xcc* strains. The results show that wild‐type and CΔflp induced similar levels of electrolyte leakage, whereas Δflp and the *hrpX* mutant induced much lower levels at 8 and 16 h, compared to the wild‐type (Fig. 7A). However, at 24 h post‐inoculation, it appeared that Δflp generated a very similar to the wild‐type and CΔflp strains but the *hrpX* mutant still retained lower levels (Fig. 7A).

In order to confirm Flp influence on T3SS *in planta* was due to modulation of *hrpX* expression, we tested an *flp* mutant that constitutively expressed *hrpX* in several HR induction assays. To achieve this, the entire ORF and Shine–Dalgarno (SD) sequence of the *hrpX* gene was introduced into the pLARF3 plasmid (under the control of the *lacZ* promoter) to generate the p3X construct (Huang *et al.*, 2009). This construct was introduced into the Δflp mutant to obtain the Δflp/p3X strain. Importantly, the Δflp/p3X strain constitutively expressing *hrpX* retained the ability to induce HR (Fig. 7A).

Callose deposition is required for disease resistance against many pathogens, including *Xcc*. It has been shown previously that *Xcc* induces defence responses in host plant *Arabidopsis* in a T3SS‐dependent manner (Rong *et al.*, 2010). Therefore, to examine potential differences in defence response we monitored callose deposition in the wild‐type, Δflp, CΔflp, Δflp/p3X and *hrpX* mutant strains. As before, we inoculated wild‐type, Δflp, CΔflp, Δflp/p3X and *hrpX* strains into cabbage leaves using the infiltration method (see Experimental procedures). Subsequently, leaves were collected at 20, 40 and 60 h post‐infiltration and callose disposition was examined (see Experimental procedures). Like HR induction the wild‐type, CΔflp and Δflp/p3X showed similar callose disposition across the leaves tested (Fig. 7B). The Δflp showed reduced levels of callose disposition compared to the wild‐type but the level, which was greater than what the *hrpX* produced, was restored to the wild‐type by the constitutive expression of *hrpX* (Fig. 7B).

To determine if growth *in planta* contributes to the HR induction and plant defence phenotypes observed, we determined the growth of strains. We examined the growth of wild‐type, Δflp, CΔflp, Δflp/p3X and *hrpX* strains following inoculation into cabbage by leaf‐cutting methods and recorded the growth variation within 10 days post‐inoculation (see Experimental procedures). The results show that the Δflp and Δflp/p3X have slower growth *in planta* compared to wild‐type and CΔflp strains (Fig. 7C). Notably, a comparison between Δflp and Δflp/p3X growth profiles showed that the latter was slower initially in colonization, which might be due to the deregulation of HrpX in this strain. Conversely, the *hrpX* mutant grew at a similar growth rate to the wild‐type and complementary strain CΔflp strains, indicating that HrpX is not initially required for host colonization.

These results together indicate that a constitutive expression of *hrpX* could restore T3SS, HR and host plant defence induction ability of the Δflp strain, which is consistent with the idea that Flp regulates T3SS by modulating the expression of *hrpX in planta*. Furthermore, Flp appears to be important for plant colonization.

### Flp influences previously uncharacterized virulence factors independently of HrpX regulation in *Xcc*

The data presented above demonstrate that Flp regulates *hrpX* by directly interacting with the promoter and therefore has an impact on T3SS. However, results from the transcriptome profile and *hrpX* complementation of the Δflp strain suggest that Flp plays a greater role in virulence than just through regulation by HrpX/T3SS.

**Figure 8:**
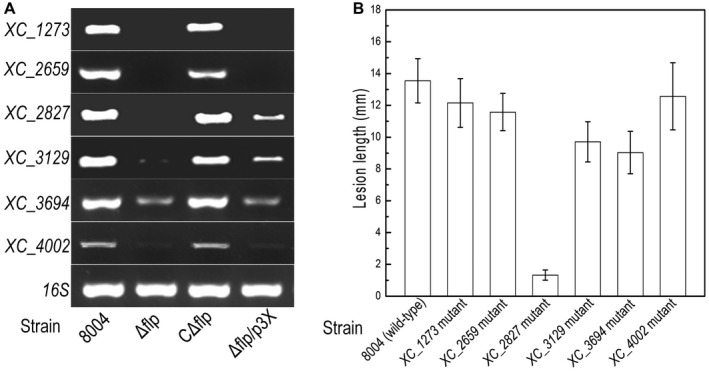
Flp influences previously uncharacterized virulence factors independently of HrpX regulation in *Xanthomonas campestris* pv. *campestris* (*Xcc)*. (A) Semi‐quantitative RT‐PCR analysis of expression of the selected genes in different *Xcc* strains: wild‐type 8004, Δflp, CΔflp and Δflp/p3X. (B) Mean lesion lengths caused by different *Xcc* mutant strains (1273nk, 2659nk, 2827nk, 3129nk, 3694nk, 4002nk) on cabbage leaves. Lesion lengths were measured at 10 days post‐inoculation. Values for means and standard deviation (SD) from 30 inoculated leaves in one experiment are indicated. The experiment was repeated three times.

To confirm this idea we compared the transcriptome profile of the Δflp strain (generated in this study) and the *hrpX* mutant strain (generated in Jiang *et al.*, 2018), which identified a set of target genes that appeared to be regulated by Flp and not HrpX. To confirm this we examined the expression of *XC_1273*, *XC_2659*, *XC_2827*, *XC_3129*, *XC_3694* and *XC_4002* by RT‐PCR by extracting RNA from wild‐type, Δflp, CΔflp and Δflp/p3X cultured in XVM2, respectively. The RT‐PCR results confirmed that expression of these selected genes was consistent with the data from the transcriptome analyses and appeared to be under the regulation of Flp (Fig. 8A). Furthermore, the expression of these genes was restored to wild‐type levels by *in trans* expression of *flp* in the Δflp mutant background (CΔflp) (Fig. 8A). Interestingly, the expression levels of genes (*XC_1273*, *XC_2659*, *XC_3694*, *XC_4002*) were not restored to wild‐type levels in the Δflp mutant background expressing *hrpX* (Fig. 8A). This suggests that genes are under the control of Flp and not HrpX.

The role of the Flp‐regulated genes in virulence to Chinese cabbage was tested by the use of a panel of insertional mutants (Table S1). The virulence of each mutant was tested by measurement of the lesion length after bacteria that were introduced into the vascular system of Chinese cabbage by leaf clipping. Mutation of three genes (*XC_2827*, *XC_3129*, *XC_3694*) gave a significant reduction in virulence (*P* < 0.05 by Student's *t*‐test) (Fig. 8B). Nevertheless, the remaining genes (*XC_1273*, *XC_2659*, *XC_4002*) showed no difference to the wild‐type strain (Fig. 8B). Despite a significant impact on virulence, none of the genes led to a complete loss of virulence. Many of these genes had not been previously associated with *Xcc* virulence.

This subset of new virulence factors for *Xcc* includes outer membrane protein (XC_3694) and inner membrane protein (XC_3129), both under the control of Flp. Interestingly, many of these proteins have homologues in other plant‐associated bacteria, including other *Xanthomonas* species and *Stenotrophomonas*.

## Discussion

Fis protein was initially identified as a factor for inversion stimulation of the homologous Hin and Gin site‐specific DNA recombinases of *E. coli* (Kahmann *et al.*, 1985). Subsequently, its diverse roles have been described, including roles in regulating bacterial virulence factors and optimizing bacterial adaptation to various environments. Fis is an abundant bacterial nucleoid‐associated protein that influences DNA topology by directly binding and bending DNA (Dillon and Dorman, 2001). It has been suggested that Fis alteration of DNA can occur in multiple tandem sites in a non‐random fashion (Schneider *et al*., 2001; Kahramanoglou *et al*., 2011). Fis has been shown to display a preference for binding to regions upstream of ORFs which can influence gene expression (Kahramanoglou *et al*., 2011). In addition, Fis can also directly activate and repress transcription at promoters by interacting with RNA polymerase (Browning *et al.*, 2010). Despite extensive studies showing that Fis serves as a global transcription factor that activates a diverse range of virulence functions, including quorum sensing, capsule production, adhesion and type III secretion in many mammalian pathogens, e.g. pathogenic *E. coli* (Falconi *et al.*, 2001; Goldberg *et al.*, 2001), *Shigella flexneri* (Falconi *et al.*, 2001), *Salmonella* *enterica* serovar Typhimurium (Kelly *et al.*, 2004) and *Yersinia pseudotuberculosis* (Green *et al.*, 2016), few works have been carried out in plant bacterial pathogens. Additionally, no such protein has been identified or characterized in *Xcc* or other bacteria from the *Xanthomonas* genus. In this study, we identified the ORF *XC_0520* from *Xcc* that encodes a small protein with 40% amino acid sequence identity to the Fis protein characterized in other bacterial species. This Fis‐like protein (or Flp) is identical in all three sequenced *Xcc* strains (8004, ATCC33913 and B100). Here, the deletion of Flp in *Xcc* caused a series of changes in virulence and HR‐associated phenotypes. Although virulence factor regulation by Fis has been seen in other bacterial plant pathogens such as *Dickeya dadantii* (Ouafa *et al*., 2012) and *Dickeya zeae* (Lv *et al*., 2018), no role has been attributed to its regulation of HR, suggesting a differentiate role in *Xcc*.

Our transcriptome analysis revealed that expression of T3SS genes, including master regulator HrpX, was down‐regulated in a *flp* mutant. We therefore investigated the regulatory effect of Flp on *hrp* genes. Using GUS‐based reporter plasmids, electrophoretic mobility shift and *in vitro* transcription assays, we demonstrated that Flp regulates the expression of *hrp* genes and T3SS by controlling the expression of *hrpX* directly. In *Xanthomonas* spp., the *hrp* genes are highly conserved and comprise more than 20 genes. The expression of *hrp* genes is mainly controlled by two key regulators, HrpG and HrpX. HrpG and HrpX form a regulatory cascade: HrpG regulates the expression of *hrpX* and HrpX then activates the expression of other *hrp* genes as well as some effector genes (Huang *et al.*, 2009; Koebnik *et al.*, 2006; Wengelnik *et al.*, 1996; Wengelnik and Bonas, 1996). Besides HrpG and HrpX, several regulators have been identified as being involved in the regulation of the expression of *hrp* genes. However, these regulators and their regulatory mechanisms are distinct in different *Xanthomonas* species, e.g. a histone‐like nucleoid‐structuring (H‐NS) protein XrvC and a LysR‐type transcriptional regulator GamR directly control the transcription of both *hrpG* and *hrpX* in *Xanthomonas oryzae* pv. *oryzae* (Liu *et al.*, 2016; Rashid *et al.*, 2016), a T3SS component HrcT positively regulates the expression of *hrpX* via binding to its promoter in *X. oryzae* pv. *oryzicola* (Liu *et al.*, 2014), and the zinc uptake regulator Zur and a GntR family transcriptional regulator HpaR1 indirectly control the expression of *hrp* genes via HrpX and HrpG, respectively, in *Xcc* (An *et al.*, 2011; Huang *et al.*, 2009). Here our experimental evidences suggest a novel regulatory pathway control T3SS in *Xcc*. However, our analysis cannot eliminate the possibility that Flp may interact with multiple ORFs to control the transcription of *hrp* genes. Furthermore, given that Flp functions as a global regulator, illustrated by our global transcriptional analysis, it is possible that Flp directly/indirectly regulates *hrp* expression via other avenues.

Our transcriptome analysis also demonstrated that Flp affects the expression of a series of other genes, such as flagellar and pilus biosynthesis, nutrition transport, stress response, amino acid and cofactor biosynthesis, galactose and starch metabolism. These genes are unlikely to be under the control of HrpX that contribute to virulence and other phenotypes such as motility and cellular stress response. This is supported by the fact that expression of HrpX in the Flp mutant cannot rescue virulence *in planta* (Fig. 1). Importantly, the transcriptome data, as well as RT‐PCR, mutation and pathogenicity experiments, unveiled a range of previously uncharacterized proteins, e.g. XC_3694 (putative outer membrane protein) and XC_3129 (putative inner membrane protein), which are required for full virulence in *Xcc*. These virulence factors appear to be regulated by a Flp‐dependent and no HrpX‐dependent mechanism. However, whether Flp have directly regulatory effects on these proteins remains unknown. Given many of them have homologues in other plant pathogens, the roles of the novel virulence factors in the pathogenicity of *Xcc* merit further investigation.

Previous studies in various bacteria have shown that Fis plays a pleiotropic role in bacterial virulence (Duprey *et al*., 2014). Our data further illustrate this in *Xcc* as Flp has a role in regulating HrpX and T3SS but also in a range of previously uncharacterized virulence factors that appear to be regulated independently of HrpX. Additionally, we demonstrated that Flp is required for swimming and swarming motility, stress tolerance, and extracellular polysaccharide and extracellular enzymes (cellulase and amylase) production. Further studies are needed to examine the role of Flp in the regulation of these phenotypes but this suggests several other questions need to be addressed: What are the environmental cues that activate the expression and activity of Flp? What genes are directly regulated by Flp? Does Flp have a conserved binding site? Does Flp regulate a different set of genes during plant colonization? Does Flp modulate the activity of HrpG directly? How does Flp affect gene expression of the new genes identified as *Xcc* virulence?

Our work demonstrates the complexity of the signalling pathways involved in the regulation of virulence in *Xcc* and describes Flp, a Fis‐like protein, an extensive regulator that controls *hrp* gene expression, HR induction and pathogenesis. The characterization of virulence protein regulators such as Flp is required to develop strategies for disease control in plant pathogens such as *Xcc*.

## Experimental Procedures

### Bacterial strains, plasmids and growth conditions

The bacterial strains and plasmids used in this work are listed in Table S1. *Escherichia coli* strains were cultured in Luria Bertani medium (Miller, 1972) at 37 °C. *Xcc* strains were cultured in NYG medium (Daniels *et al.*, 1984), NY medium (NYG medium but without glycerol), and the mimic medium XVM2 (Wengelnik and Bonas, 1996) at 28 °C and 200 rpm. Antibiotics were used according to the concentrations as required: kanamycin at 25 µg/mL, rifampicin at 50 µg/mL, ampicillin at 100 µg/mL, spectinomycin at 50 µg/mL and Tet at 5 µg/mL for *Xcc* strains and 15 µg/mL for *E. coli* strains.

### Nucleus acid manipulations

The nucleic acid manipulations followed the procedures described by Sambrook *et al.* (1989). Conjugation between the *Xcc* and *E. coli* strains was performed as described by Turner *et al.* (1985). The restriction endonucleases, T4 DNA ligase and *Pfu* polymerase were provided by Promega (Shanghai, China). The total RNAs were extracted from the cultures of the *Xcc* strains with a total‐RNA extraction kit (Invitrogen, Waltham, MA, USA) and cDNA generated using a cDNA synthesis kit (Invitrogen). These kits were used with reference to the manufacturer's instructions. For semi‐quantitative RT‐PCR, the obtained cDNA was diluted and used as a template with selected primers for target genes (Table S3).

### Construction of mutant strains

In order to construct the in‐frame deleted mutant of *flp* (*XC_0520*), 333 bp (*Eco*RI and *Bam*HI) of the upstream and 339 bp (*Bam*H1 and *Hin*dIII) of the downstream sequences of the *flp* gene were amplified by PCR using the relevant primers (Table S3). After being digested by restriction enzymes, these DNA fragments were fused with the suicide plasmid pK18*mobsacB* (Schäfer *et al.*, 1994) and transformed into *E. coli* DH5α. The acquired recombined plasmid was introduced into *Xcc* with the help of plasmid pRK2073 (Table S1). The original ORF of *XC_0520* will be deleted from genomes through allelic exchange and homologous recombination.

To complement the *flp* deletion mutant, an 297‐bp DNA fragment of the *flp* gene coding sequence was PCR‐amplified from *Xcc* strain 8004 and inserted into the pLAFR3 vector at the *Bam*HI/*Hin*dIII restriction site, creating the plasmid pLCflp (Table S1). This plasmid was introduced into the mutant by triparental mating.

### Pathogenicity tests, HR assays, leakage assays and in‐plant growth curve

The virulence of the *Xcc* strain to the host cabbage plant (*Brassica oleracea* 'Jingfeng No. 1') was tested by the leaf‐clipping method (Wang *et al.*, 2007). Cabbage seedlings were planted and grown in the greenhouse for 30 days and the leaves were used for inoculation. *Xcc* strains, collected from overnight culture, were washed and adjusted to the same final density (OD_600_ = 0.6, approximately 1 × 10^9^ CFU/mL). The bacterial resuspension was then diluted to 1 × 10^6^ CFU/mL. The lesion and symptoms were measured 10 days post‐inoculation (Dow *et al.*, 2003).

HR was tested on pepper leaves (*Capsicum annuum* cv. ECW‐10R) as previously described (Li *et al.*, 2014). Briefly, bacteria suspensions (1 × 10^8^ CFU/mL) were infiltrated into the abaxial side of the pepper leaves. These inoculated plants were kept in the greenhouse at 28 °C to observe the HR symptoms and gauge conductivity. For conductivity measurements, samples (leaf discs of 0.4 cm^2^) were collected using a hole‐puncher. These acquired samples were soaked in 10 mL ultrapure water with shaking at 200 rpm. The leaf discs were then removed and the conductivity of water was measured with a DDS‐307A conductivity meter.

Bacterial *in planta* growth was estimated as previously described (Li *et al.*, 2014). Briefly, *Xcc* strains were inoculated onto cabbage leaves using leaf clippings. At intervals of 24 h, four clipped leaves from every group of inoculated plants were collected and homogenized. Homogenate was serially diluted using NYG medium and 100 μL dilution was used for spread plate counting (NYG medium). The amounts of bacteria were calculated after 2 days of incubation.

### Callose deposition assay

Callose deposition assay was performed on cabbage leaves as recently described by Hamdoun *et al.* (2018). Thirty‐day‐old cabbage leaves were inoculated with *Xcc* strains by infiltration (detailed above). At intervals of 20 h, the leaves were collected and individually soaked in 70% ethanol for 2 h, then 50% ethanol for 2 h and finally sterile distilled water for 2 h. When the chlorophyll was completely leached, the leaves were stained in 0.01% aniline blue. Callose deposition was observed using an Olympus BH‐2 epifluorescent microscope.

### GUS activity assays

GUS activity in bacterial strains was measured as described by Henderson *et al.* (1985). Wild‐type and mutant strains harbouring reporter plasmids were cultured in XVM2 media at 28 °C for 8 h. Bacteria cells were collected by centrifugation and resuspended in 375 μL of 1 mM *p*‐nitrophenyl‐β‐d‐glucuronide extraction buffer (50 mM sodium dihydrogen phosphate, 0.1% Triton X‐100 and 10 mM *β*‐mercaptoethanol, pH 7.2) and incubated at 37 °C for 10 min, and then terminated by 200 mL of 2.5 M 2‐amino‐2‐methyl‐1,3‐propanediol. Enzyme assays were carried out in triplicate from at least three independent cultures.

### Protein manipulation

In order to obtain purified Flp protein, the *flp* gene was cloned and introduced into the expression vector pQE30 [harbouring a *lac* promoter and a ribosome binding site [RBS] in front of the multiple cloning site (MCS)]. The Flp protein was expressed and purified with the methods modified from An *et al.* (2011). To remove imidazole, 6 × His‐tagged Flp was dialysed against 200 volumes of Tris‐HCl buffer [10 mM Tris‐HCl (pH 8.0) and 1 mM dithiothreitol (DTT)] at 4 °C.

### Electrophoretic mobility shift assays

The method that was deployed for EMSA assays was modified from that previously described by Su *et al.* (2016). Here fragments of the gene encoding HrpX were PCR amplified with the primers described in Table S3. These DNA fragments were labelled with FAM at the 5ʹ terminal. The Flp protein and selected DNA fragments were mixed with the binding buffer and incubated for 20 min at 28 °C. The reaction samples were loaded onto a 6% polyacrylamide‐Tris‐glycine‐EDTA gel. Electrophoresis was performed in TGE buffer (pH 7.6) and visualized using an autoradiograph.

### *In vitro* transcription assays

*In vitro* transcription assays were performed as previously described (Su *et al.*, 2016). Promoter sequence fragments (311‐bp) of *hrpX* were acquired using PCR (see Table S3). 6 × His‐tagged Flp protein and DNA fragments were incubated for 30 min at room temperature in transcription buffer. Then, a NTP mixture (250 μM each of ATP, CTP and GTP; 250 μM biotin‐16‐UTP) and 0.5 U of *E. coli* RNA polymerase holoenzyme (New England BioLabs, Ipswich, MA, USA) were added to initiate transcription. After incubation at 28 °C for 30 min, the reactions were terminated and the transcription products were analysed by electrophoresis. The transcripts obtained were visualized using a phosphor imager screen (GE AI600).

### Stress tolerance assay

The minimal inhibitory concentration (MIC) method (Su *et al.*, 2016) was employed to test the sensitivity of the *Xcc* strains to several environmental stresses, including sodium dodecyl sulphate (SDS), hydrogen peroxide (H_2_O_2_), hyperosmosis (NaCl), phenol and heavy metal salts (CdCl_2_, CuSO_4_) stress. Briefly, *Xcc* strains were cultured overnight and diluted to an OD_600_ of 0.1, then 100 μL of the diluted culture was plated on NYG plates supplemented with different concentrations of each reagent, respectively. The surviving colonies on the plates were counted after 3 days of incubation at 28 °C.

### Exopolysaccharide and extracellular enzymes assays

Exopolysaccharide (EPS) and extracellular enzymes assays were performed as previously described (Su *et al.*, 2016; Tang *et al.*, 1991). To estimate EPS production, *Xcc* strains were inoculated into 100 mL NY liquid medium containing glucose (2% w/v) at 28 °C, 200 rpm for 5 days. EPS was precipitated from the culture supernatant with ethanol and dried at 55 °C using an oven and weighed. For quantitative estimation of the activity of the extracellular enzymes endoglucanase (cellulase) and amylase, *Xcc* strains were cultured in NYG medium for 12 h. For endoglucanase, 10 μL of enzyme‐containing extracts was added to 200 μL of indicator buffer containing 1% (wt/vol) carboxymethylcellulose (CMC, Sangon, Shangshai, China) as the substrate. The reactions were carried out for 30 min at 28 °C. The released reducing sugars were measured as d‐glucose equivalents, as described by Miller (1959). One unit (U) of the endoglucanase activity was defined as the amount of enzyme releasing 1 μmol of reducing sugar per minute. Amylase activity quantification was conducted in the same way as for the endoglucanase measurement, except that the substrate 1% (wt/vol) CMC was replaced by 1% (wt/vol) starch solution.

### Transcriptome analysis of the Flp mutant

Transcriptome analysis were performed as previously described (Cui *et al.*, 2018). Briefly, RNA was prepared from cell culture of OD_600_ = 0.6. Contaminating genomic DNA was removed using RNase‐free DNase I. After the quantity determination and quality assessment, total RNA was sent to Novogene (Beijing, China) for library construction and strand‐specific RNA sequencing. Sequencing libraries were generated using a NEBNext Ultra™ Directional RNA Library Prep Kit for Illumina (New England BioLabs), and sequenced on an Illumina (CA, USA) HiSeq 2000 platform. Clean reads were mapped to the reference genome and the RPKM (reads per kilobase per million mapped reads) method was used to calculate the gene expression levels. False discovery rate FDR ≤ 0.05 and |log_2_FC| (log_2_ of the fold changes) ≥1 were considered for differentially expressed genes.

### ChIP assay

ChIP assay was performed as previously described with minor modifications (Liu *et al.*, 2019). In brief, a strain producing an Flp protein fused with 3 × Flag‐tag (3 × Flag::Flp) at the N‐terminus of Flp was first constructed. To do this, a DNA fragment encoding Flp fused with 3 × Flag peptide was obtained using PCR with the primer set Fflag‐F/R (Table S3) and cloned into the *Bam*HI/*Hin*dIII sites of the vector pLAFR3. The acquired recombinant plasmid pFlp‐Flag was introduced into *Xcc* flp deletion strain Δflp, resulting in strain Δflp/pFlp‐Flag. *Xcc* wild‐type strain 8004 containing the empty vector pLAFR3 (8004/pLAFR3) was used as a negative control. *Xcc* strains were grown in XVM2 medium for 12 h and cross‐linked using formaldehyde. Bacterial cells were collected and then lysed by sonication. For each ChIP sample, 50 μL of anti‐Flag (agarose conjugated) was added to the bacterial lysates and incubated overnight. Unbound DNA fragments were washed and the bound DNA fragments and proteins were eluted by 0.25 M glycine (pH 2.5).

## Supporting information


**Fig. S1** The homology of Flp and its position in evolution. (A) Sequence alignments between Flp and Fis proteins in *Yersinia pseudotuberculosis*, *Dickeya zeae* and *E. coli*. The sequences of these proteins were acquired from NCBI website and the alignment was proceeded with the software NTI Vector. (B) The position of Flp in evolutionary tree. A series of Fis family proteins were acquired from NCBI, and the evolutionary tree of these proteins was compiled with MEGA6.0. The position on the branches of the tree indicated the distance in evolution.

**Table S1** Bacterial strains and plasmids used in this work. Note: ^a^Rif^r^, Kan^r^, Tet^r^ and Spc^r^ indicate resistance to rifampicin, kanamycin, tetracycline and spectinomycin, respectively.

**Table S2** Genes expressed by the Δflp mutant strain when grown in XVM2.

**Table S3** Primers used in this study.
