# Supplementary material for: Flp, a Fis‐like protein, contributes to the regulation of type III secretion and virulence processes in the phytopathogen Xanthomonas campestris pv. campestris
Source: Mol Plant Pathol. 2019 May 14;20(8):1119–33. doi: 10.1111/mpp.12818 (PMC6640185; doi:10.1111/mpp.12818)
Supplement: Supplementary file 1 — Fig. S1 The homology of Flp and its position in evolution. (A) Sequence alignments between Flp and Fis proteins in Yersinia pseudotuberculosis, Dickeya zeae and E. coli. The sequences of these proteins were acquired from NCBI website and the alignment was proceeded with the software NTI Vector. (B) The position of Flp in evolutionary tree. A series of Fis family proteins were acquired from NCBI, and the evolutionary tree of these proteins was compiled with MEGA6.0. The position on the branches of the tree indicated the distance in evolution. [file MPP-20-1119-s001.pdf]

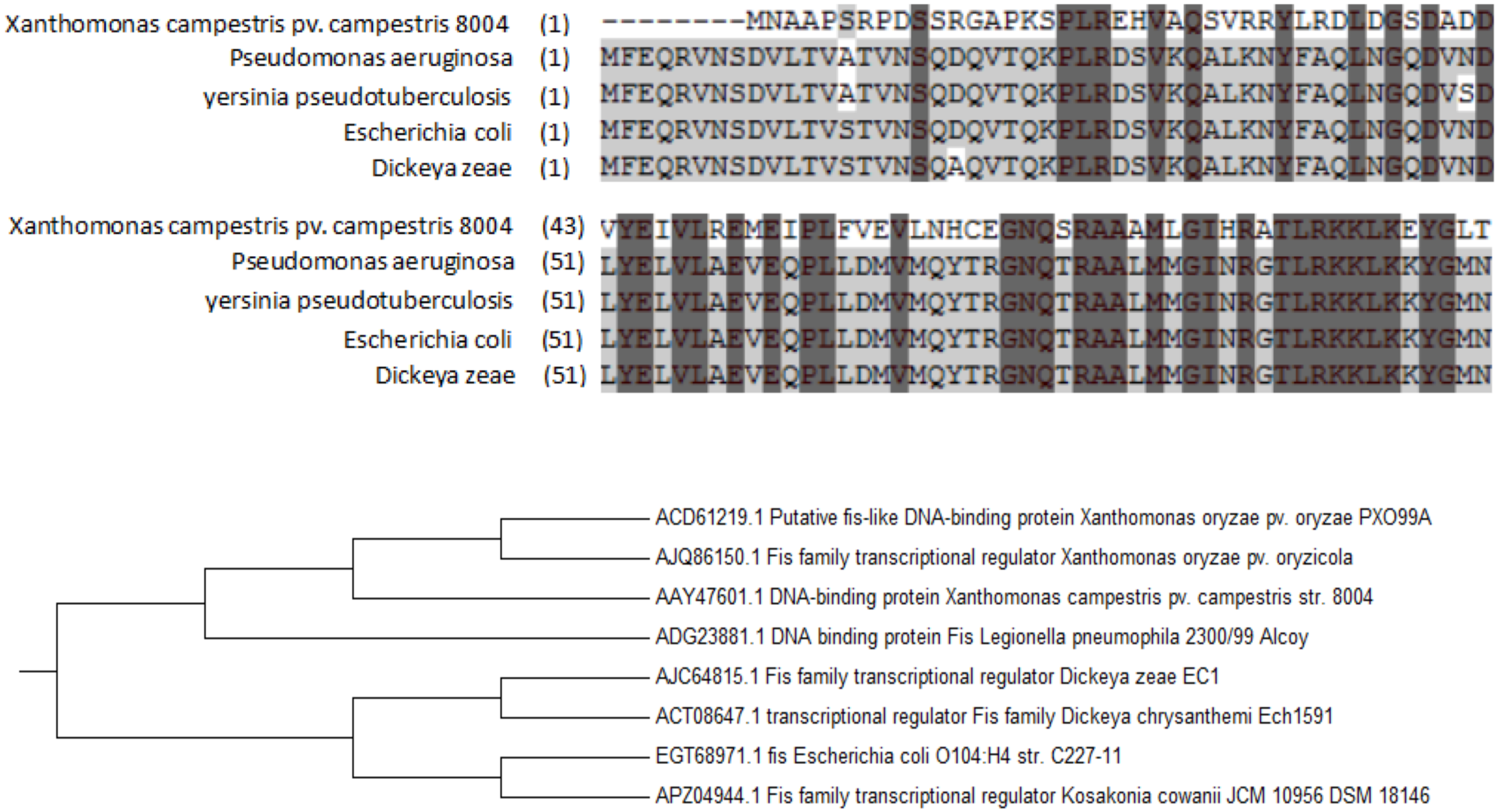

Xanthomonas campestris pv. campestris 8004 (1) --------MNAAPSRPDSSRGAPKSPLREHVAQSVRRYLRDLDGSDADD
Pseudomonas aeruginosa (1) MFEQRVNSDVLTVATVNSQDQVTQKPLRDSVKQALKNYFAQLNGQDVND
yersinia pseudotuberculosis (1) MFEQRVNSDVLTVATVNSQDQVTQKPLRDSVKQALKNYFAQLNGQDVSD
Escherichia coli (1) MFEQRVNSDVLTVSTVNSQDQVTQKPLRDSVKQALKNYFAQLNGQDVND
Dickeya zeae (1) MFEQRVNSDVLTVSTVNSQAQVTQKPLRDSVKQALKNYFAQLNGQDVND
Xanthomonas campestris pv. campestris 8004 (43) VYEIVLREMEIPLFVEVLNHCEGNQSRAAAMLGIHRATLRKKLKEYGLT
Pseudomonas aeruginosa (51) LYELVLAEVEQPLLDMVMQYTRGNQTRAALMMGINRGTLRKKLKKYGMN
yersinia pseudotuberculosis (51) LYELVLAEVEQPLLDMVMQYTRGNQTRAALMMGINRGTLRKKLKKYGMN
Escherichia coli (51) LYELVLAEVEQPLLDMVMQYTRGNQTRAALMMGINRGTLRKKLKKYGMN
Dickeya zeae (51) LYELVLAEVEQPLLDMVMQYTRGNQTRAALMMGINRGTLRKKLKKYGMN
ACD61219.1 Putative fis-like DNA-binding protein Xanthomonas oryzae pv. oryzae PXO99A
AJQ86150.1 Fis family transcriptional regulator Xanthomonas oryzae pv. oryzicola
AAY47601.1 DNA-binding protein Xanthomonas campestris pv. campestris str. 8004
ADG23881.1 DNA binding protein Fis Legionella pneumophila 2300/99 Alcoy
AJC64815.1 Fis family transcriptional regulator Dickeya zeae EC1
ACT08647.1 transcriptional regulator Fis family Dickeya chrysanthemi Ech1591
EGT68971.1 fis Escherichia coli O104:H4 str. C227-11
APZ04944.1 Fis family transcriptional regulator Kosakonia cowanii JCM 10956 DSM 18146

Fig. S1
