# Supplementary material for: Flp, a Fis‐like protein, contributes to the regulation of type III secretion and virulence processes in the phytopathogen Xanthomonas campestris pv. campestris
Source: Mol Plant Pathol. 2019 May 14;20(8):1119–33. doi: 10.1111/mpp.12818 (PMC6640185; doi:10.1111/mpp.12818)
Supplement: Supplementary file 2 — Table S1 Bacterial strains and plasmids used in this work. Note: aRifr, Kanr, Tetr and Spcr indicate resistance to rifampicin, kanamycin, tetracycline and spectinomycin, respectively. [file MPP-20-1119-s002.docx]

**Table S1**. Bacterial strains and plasmids used in this work

| Strains or plasmids | Relevant characteristics | Reference or source |
| --- | --- | --- |
| *E. coli* strains |  |  |
| DH5α | Φ80△*lacZM*15 *recA1 endA1 deoR* | Gibco BRL, Life Technologies |
| M15 | *lac ara gal mtl recA1 uvr1* [pREP4 *lacI* Kan^r^] | Novagen, Germany |
| M15/pQE30-flp | M15 harbouring recombinant pQE30-flp | This work |
| *Xanthomonas campestris* pv. *campestris* strains |  |  |
| 8004 | Wild-type strain, Rif^r^ | Daniels *et al.*, 1984 |
| 0520nk | As 8004, but *XC_0520*::pK18*mob*, non-polar effect. Rif^r^ Kan^r^. | Author’s lab collection |
| Δflp | As 8004, but flp gene (*XC_0520*) deleted, non-polar effect. Rif^r^ | This work |
| C∆flp | ∆flp harboring the recombinant plasmid pLCflp. Rif^r^ Tet^r^ | This work |
| 8004/p3X | 8004 harboring the recombinant plasmid p3X. Rif^r^ Tet^r^ | This work |
| Δflp /p3X | ∆flp harboring the recombinant plasmid p3X. Rif^r^ Tet^r^ | This work |
| ΔhrpX | As 8004, but *hrpX* deleted. Rif^r^ | Author’s lab collection |
| 1273nk | As 8004, but *XC_1273*::pK18*mob*, non-polar effect. Rif^r^ Kan^r^. | Author’s lab collection |
| 2659nk | As 8004, but *XC_2659*::pK18*mob*, non-polar effect. Rif^r^ Kan^r^. | Author’s lab collection |
| 2827nk | As 8004, but *XC_2827*::pK18*mob*, non-polar effect. Rif^r^ Kan^r^. | Author’s lab collection |
| 3129nk | As 8004, but *XC_3129*::pK18*mob*, non-polar effect. Rif^r^ Kan^r^. | Author’s lab collection |
| 3694nk | As 8004, but *XC_3694*::pK18*mob*, non-polar effect. Rif^r^ Kan^r^. | Author’s lab collection |
| 4002nk | As 8004, but *XC_4002*::pK18*mob*, non-polar effect. Rif^r^ Kan^r^. | Author’s lab collection |
| 8004/pGUS*hrpG* | 8004 harboring the reporter plasmid pGUS*hrpG*. Rif^r^ Tet^r^ | This work |
| 8004/pGUS*hrpX* | 8004 harboring the reporter plasmid pGUS*hrpX*. Rif^r^ Tet^r^ | This work |
| 8004/pGUS*hrpB* | 8004 harboring the reporter plasmid pGUS*hrpB*. Rif^r^ Tet^r^ | This work |
| 8004/pGUS*hrpC* | 8004 harboring the reporter plasmid pGUS*hrpC*. Rif^r^ Tet^r^ | This work |
| 8004/pGUS*hrpF* | 8004 harboring the reporter plasmid pGUS*hrpF*. Rif^r^ Tet^r^ | This work |
| Δflp/pGUS*hrpG* | Δflp harboring the reporter plasmid pGUS*hrpG*. Rif^r^ Tet^r^ | This work |
| Δflp/pGUS*hrpX* | Δflp harboring the reporter plasmid pGUS*hrpX*. Rif^r^ Tet^r^ | This work |
| Δflp/pGUS*hrpB* | Δflp harboring the reporter plasmid pGUS*hrpB*. Rif^r^ Tet^r^ | This work |
| Δflp/pGUS*hrpC* | Δflp harboring the reporter plasmid pGUS*hrpC*. Rif^r^ Tet^r^ | This work |
| Δflp/pGUS*hrpF* | Δflp harboring the reporter plasmid pGUS*hrpF*. Rif^r^ Tet^r^ | This work |
| 8004/pLAFR3 | 8004 harboring the vector pLAFR3. Rif^r^ Tet^r^ | This work |
| Δflp(pFlp-Flag) | Δflp harboring the plasmid pFlp*-*Flag. Rif^r^ Tet^r^ | This work |
| Plasmids |  |  |
| pLAFR3 | Broad host range cloning vector. Tet^r^ | Staskawicz *et al*.,1987 |
| pLCflp | pLAFR3 containing an 297-bp DNA fragment of the *flp* gene (*XC_0520*) of *Xcc* strain. Tet^r^ | This work |
| pFlp-Flag | pLAFR3 containing 3×Flag-tag fused flp (3×Flag::Flp). Tet^r^ | This work |
| p3X | pLARF3 plasmid containing the entire ORF and SD sequence of *hrpX* gene | Huang *et al*., 2009 |
| pRK2073 | Helper plasmid, Tra^+^, Mob^+^, ColE1, Spc^r^. | Leong *et al*.,1982 |
| pK18*mob* | pUC18 derivative, *lacZα* Kan^r^, *mob* site. Suicide plasmid in *Xcc*. | Schäfer *et al.*, 1994 |
| pK18*mobsacB* | pUC18 derivative, *lacZα*, *sacB*, Kan^r^, *mob* site. Allelic exchange vector (Suicidal vector carrying *sacB* gene for mutagenesis). | Schäfer *et al*., 1994 |
| pKSΔflp | pK18*mobsacB* containing fragments flanking *flp* gene. Kan^r^ | This work |
| pQE-30 | Expression vector, allow the production of fusion proteins containing amino terminal 6×His-tagged sequences.Ampr | Qiagen |
| pQE30-flp | pQE-30a containing an 270-bp fragment of *flp* gene. | This work |
| pGUS*hrpG* | pLAFR6 containing an *hrpG* promoter-*gusA* fusion fragment. Tet^r^ | Author’s lab collection |
| pGUS*hrpX* | pLAFR6 containing an *hrpX* operon promoter-*gusA* fusion fragment. Tet^r^ | Author’s lab collection |
| pGUS*hrpB* | pLAFR6 containing an *hrpB* operon promoter-*gusA* fusion fragment. Tet^r^ | Author’s lab collection |
| pGUS*hrpC* | pLAFR6 containing an *hrpC* operon promoter-*gusA* fusion fragment. Tet^r^ | Author’s lab collection |
| pGUS*hrpF* | pLAFR6 containing an *hrpF* operon promoter-*gusA* fusion fragment. Tet^r^ | Author’s lab collection |

^a^Rif^r^, Kan^r^, Tet^r^, Ampr and Spc^r^ indicate resistance to rifampicin, kanamycin, tetracycline, ampicillin and spectinomycin, respectively.

**References**

Daniels, M.J., Barber, C.E., Turner, P.C., Sawczyc, M.K., Byrde, R.J.W., and Fielding, A.H. (1984) Cloning of genes involved in pathogenicity of *Xanthomonas campestris* pv. *campestris* using the broad host range cosmid pLAFR1. *EMBO J.* 3, 3323–3328.

Huang, D.L., Tang, D.J., Liao, Q., Li, X.Q., He, Y.Q., Feng, J.X., Jiang, B.L., Lu, G.T. and Tang, J.L. (2009) The Zur of *Xanthomonas campestris* is involved in hypersensitive response and positively regulates the expression of the *hrp* cluster via *hrpX* but not *hrpG*. *Mol. Plant Microbe Interact.* 22, 321−329.

Leong, S.A., Ditta, G.S., and Helinski, D.R. (1982) Heme biosynthesis in *Rhizobium*. Identification of a cloned gene coding for delta-aminolevulinic acid synthetase from *Rhizobium meliloti*. *J. Biol. Chem.* 257, 8724–8730.

Schäfer, A., Tauch, A., Jäger, W., Kalinowski, J., Thierbach, G., and Pühler, A. (1994) Small mobilizable multi-purpose cloning vectors derived from the *Escherichia coli* plasmids pK18 and pK19: selection of defined deletions in the chromosome of *Corynebacterium glutamicum*. *Gene* 145, 69–73.

Staskawicz, B., Dahlbeck, D., Keen, N., and Napoli, C. (1987) Molecular characterization of cloned avirulence genes fromrace 0 and race 1 of *Pseudomonas syringae* pv. *glycinea*. *J. Bacteriol.* 169, 5789–5794.
