# Supplementary material for: Flp, a Fis‐like protein, contributes to the regulation of type III secretion and virulence processes in the phytopathogen Xanthomonas campestris pv. campestris
Source: Mol Plant Pathol. 2019 May 14;20(8):1119–33. doi: 10.1111/mpp.12818 (PMC6640185; doi:10.1111/mpp.12818)
Supplement: Supplementary file 3 — Table S2 Genes expressed by the Δflp mutant strain when grown in XVM2. [file MPP-20-1119-s003.docx]

Table S2. The up- and down-regulated differential expressed genes of the flp mutant ∆flp and cultured in the XVM2 medium

| Function Category | Gene_ID | Name | Annotation | Fold change |
| --- | --- | --- | --- | --- |
| Amino acids biosynthesis | *XC_0330* | *metE* | 5-methyltetrahydropteroyltriglutamate-homocysteine methyltransferase | -7.73 |
|  | *XC_2374* | *hisI* | phosphoribosyl-AMP cyclohydrolase | -4.56 |
|  | *XC_2375* | *hisF* | cyclase | -4.63 |
|  | *XC_2376* | *hisA* | phosphoribosylformimino-5-aminoimidazole carboxam | -4.18 |
|  | *XC_2377* | *hisH* | amidotransferase | -4.57 |
|  | *XC_2378* | *hisB* | imidazole glycerol phosphate dehydratase | -4.10 |
|  | *XC_3676* | *pheA* | chorismate mutase | -2.75 |
|  | *XC_1569* | *asd* | aspartate semialdehyde dehydrogenase | +2.27 |
| Biosynthesis of cofactors, prosthetic groups, carriers | *XC_1111* | *bioI* | cytochrome P450 hydroxylase | -3.59 |
|  | *XC_1169* | *pqqE* | pyrroloquinoline quinone biosynthesis protein E | -2.72 |
|  | *XC_1171* | *pqqC* | pyrroloquinoline quinone biosynthesis protein PqqC | -2.18 |
|  | *XC_2090* |  | oxygen-independent coproporphyrinogen III oxidase | -2.24 |
|  | *XC_3157* |  | hydroxylase molybdopterin-containing subunit | -2.48 |
| Cell envelope and cell structure | *XC_0416* | *mdoB* | phosphoglycerol transferase I | -2.11 |
|  | *XC_1459* | *phuR* | outer membrane hemin receptor | -2.16 |
|  | *XC_2151* |  | L-sorbosone dehydrogenase | -2.11 |
|  | *XC_2171* | *rfbD* | strX protein | -2.53 |
|  | *XC_2588* | *gtrB* | glycosyl transferase-related protein | -3.00 |
|  | *XC_3129* | *yjdB* | putative membrane-associated hydrolase | -3.95 |
|  | *XC_3513* | *murF* | UDP-N-acetylmuramoylalanyl-D-glutamyl-2, 6-diaminopimelate-D-alanyl-D-alanyl ligase | -2.14 |
|  | *XC_3515* | *ftsI* | penicillin-binding protein 3 | -2.06 |
|  | *XC_1184* | *pilH* | type IV pilus response regulator PilH | +3.07 |
|  | *XC_1185* | *pilI* | pilI | +3.04 |
|  | *XC_1187* | *pilL* | two-component system sensor histidine | +2.70 |
|  | *XC_1219* | *nahA* | beta-hexosaminidase | +2.08 |
|  | *XC_1358* | *pilT* | twitching motility protein | +2.35 |
|  | *XC_1359* | *pilU* | twitching motility protein | +2.01 |
|  | *XC_1619* | *oar* | TonB-dependent outer membrane receptor | +2.36 |
| Cellular processes | *XC_0141* |  | alpha-amylase | -2.99 |
|  | *XC_0142* | *treS* | trehalose synthase | -3.71 |
|  | *XC_0143* | *glgB1* | 1,4-alpha-glucan branching enzyme | -2.38 |
|  | *XC_0336* | *tsr* | chemotaxis protein | -2.97 |
|  | *XC_0422* | *glgB2* | 1,4-alpha-glucan branching enzyme | -2.53 |
|  | *XC_0423* | *TreZ* | maltooligosyltrehalose trehalohydrolase | -3.04 |
|  | *XC_0424* | *malQ* | 4-alpha-glucanotransferase | -2.41 |
|  | *XC_0761* | *betB* | betaine aldehyde dehydrogenase | -2.20 |
|  | *XC_2551* |  | hexosyltransferase | -2.18 |
|  | *XC_0638* | *tsr* | chemotaxis protein | +3.07 |
|  | *XC_1123* |  | alpha-trehalose-phosphate synthase | +2.53 |
|  | *XC_1413* | *mcp* | chemotaxis protein | +2.59 |
|  | *XC_1801* | *mcp* | chemotaxis protein | +2.66 |
|  | *XC_2223* | *mcp* | chemotaxis protein | +5.57 |
|  | *XC_2231* | *flgM* | negative regulator of flagellin synthesis | +5.32 |
|  | *XC_2235* | *flgC* | flagellar biosynthesis | +2.04 |
|  | *XC_2237* | *flgE* | flagellar hook protein | +3.71 |
|  | *XC_2238* | *flgF* | flagellar protein | +2.12 |
|  | *XC_2239* | *flgG* | flagellar biosynthesis | +3.14 |
|  | *XC_2240* | *flgH* | flagellar L-ring protein | +2.54 |
|  | *XC_2241* | *flgI* | flagellar P-ring protein precursor | +2.61 |
|  | *XC_2242* | *flgJ* | flagellar protein | +4.14 |
|  | *XC_2243* | *flgK* | flagellar protein | +2.87 |
|  | *XC_2244* | *flgL* | flagellar protein | +2.13 |
|  | *XC_2245* | *fliC* | flagellar protein | +9.73 |
|  | *XC_2246* | *fliD* | flagellar protein | +3.64 |
|  | *XC_2279* | *flhF* | flagellar biosynthetic protein | +2.09 |
|  | *XC_2282* | *cheY* | chemotaxis protein | +6.99 |
|  | *XC_2283* | *cheZ* | chemotaxis related protein | +7.56 |
|  | *XC_2284* | *cheA* | chemotaxis related protein | +4.83 |
|  | *XC_2297* | *motA* | MotA protein | +4.56 |
|  | *XC_2300* | *cheW* | chemotaxis protein | +3.03 |
|  | *XC_2302* | *cheY* | chemotaxis response regulator | +5.81 |
|  | *XC_2304* | *tsr* | chemotaxis protein | +2.24 |
|  | *XC_2309* | *tsr* | chemotaxis protein | +6.21 |
|  | *XC_2311* | *tsr* | chemotaxis protein | +7.62 |
|  | *XC_2315* | *mcp* | methyl-accepting chemotaxis protein | +3.56 |
|  | *XC_2316* | *tsr* | chemotaxis protein | +2.77 |
|  | *XC_2320* | *tsr* | chemotaxis protein | +17.74 |
| Central intermediary metabolism | *XC_0154* | *mhpD* | 2-keto-4-pentenoate hydratase | -4.16 |
|  | *XC_0202* | *gloA* | lactoylglutathione lyase | -2.25 |
|  | *XC_0374* | *pobB* | phenoxybenzoate dioxygenase beta subunit | -2.51 |
|  | *XC_0375* | *vanA* | vanillate O-demethylase oxygenase subunit | -2.05 |
|  | *XC_0378* | *pcaF* | beta-ketoadipyl CoA thiolase | -2.30 |
|  | *XC_0427* | *glgX1* | glycogen debranching enzyme | -2.47 |
|  | *XC_1047* | *glgX2* | glycogen debranching enzyme | -2.74 |
|  | *XC_1714* | *xsa* | xylosidase/arabinosidase | -2.22 |
|  | *XC_2325* | *pcaD* | beta-ketoadipate enol-lactone hydrolase | -3.77 |
|  | *XC_3712* | *sndH* | L-sorbosone dehydrogenase | -2.31 |
|  | *XC_0450* |  | 4-hydroxyphenylpyruvate dioxygenase | +3.09 |
|  | *XC_0452* | *hmgA* | homogentisate 1,2-dioxygenase | +2.35 |
|  | *XC_0990* | *cysH* | 3'-phosphoadenosine 5'-phosphosulfate reductase | +5.24 |
|  | *XC_0991* | *cysI* | NADPH-sulfite reductase iron-sulfur protein | +3.9 |
|  | *XC_0993* | *cysD* | ATP sulfurylase small subunit | +3.65 |
|  | *XC_1002* | *susB* | alpha-glucosidase | +2.13 |
|  | *XC_1218* |  | beta-mannosidase | +2.22 |
|  | *XC_1642* | *aglA* | alpha-glucosidase | +3.05 |
|  | *XC_2477* | *xylA* | xylose isomerase | +5.79 |
|  | *XC_2653* | *hutI* | imidazolone propionase | +2.45 |
|  | *XC_2654* | *sdeB* | atrazine chlorohydrolase | +2.19 |
|  | *XC_2657* | *hutU* | urocanate hydratase | +3.43 |
|  | *XC_3032* |  | tryptophan 2,3-dioxygenase | +2.35 |
|  | *XC_3050* | *galM* | aldose 1-epimerase | +2.01 |
|  | *XC_3054* | *lamA* | endo-1,3-beta-glucanase precursor | +2.34 |
|  | *XC_3456* | *tauD* | taurine dioxygenase | +4.40 |
|  | *XC_3487* | *amy* | alpha-amylase | +3.31 |
|  | *XC_4191* | *xylA* | xylose isomerase | +3.09 |
| Energy and carbon metabolism | *XC_0216* | *poxB* | pyruvate dehydrogenase | -2.15 |
|  | *XC_0328* | *sflA* | NADH-dependent FMN reductase | -2.04 |
|  | *XC_0377* | *gctB* | glutaconate CoA transferase subunit B | -2.21 |
|  | *XC_1385* | *yagT* | oxidoreductase | -2.01 |
|  | *XC_1386* | *yagS* | oxidoreductase | -3.22 |
|  | *XC_2188* | *fdh* | glutathione-dependent formaldehyde dehydrogenase | -3.28 |
|  | *XC_2192* | *yxnA* | glucose 1-dehydrogenase homolog | -2.56 |
|  | *XC_2572* | *cycK* | C-type cytochrome biogenesis membrane protein | -2.14 |
|  | *XC_2585* |  | dehydrogenase | -2.30 |
|  | *XC_2659* | *gcd* | glucose dehydrogenase | -7.30 |
|  | *XC_3167* |  | oxidoreductase | -2.55 |
|  | *XC_3763* | *cioB* | cyanide insensitive terminal oxidase | -3.10 |
|  | *XC_3896* | *coxD* | cytochrome C oxidase assembly factor | -2.21 |
|  | *XC_3994* |  | cytochrome B561 | -2.46 |
|  | *XC_0029* | *yahK* | alcohol dehydrogenase | +2.25 |
|  | *XC_0247* | *mls* | malate synthase | +4.22 |
|  | *XC_0248* | *aceA* | isocitrate lyase | +2.15 |
|  | *XC_0686* | *adhC* | alcohol dehydrogenase class III | +2.57 |
|  | *XC_2797* |  | short chain dehydrogenase | +3.56 |
|  | *XC_2800* | *dauE* | aklaviketone reductase | +4.02 |
|  | *XC_3307* | *mdh* | malate dehydrogenase | +3.10 |
| Fatty acid and phospholipid acid metsbolism | *XC_0035* |  | phospholipid N-methyltransferase | -2.16 |
|  | *XC_2818* |  | phospholipase | -2.56 |
|  | *XC_4263* | *cls* | cardiolipin synthetase | -2.45 |
|  | *XC_1682* | *blc* | outer membrane lipoprotein | +2.54 |
|  | *XC_3652* | *fabB* | beta-ketoacyl-[ACP] synthase I | +2.05 |
| Regulatory functions | *XC_0752* |  | transcriptional regulator lacI family | -2.06 |
|  | *XC_0848* |  | transcriptional regulator | -2.72 |
|  | *XC_2130* | *cheY* | single-domain response regulator | -2.74 |
|  | *XC_2712* | *phoU* | phosphate transport system protein | -2.19 |
|  | *XC_2973* | *mucA* | putative negative regulator of sigma E activity | -5.94 |
|  | *XC_3425* | *pcaQ* | transcriptional regulator | -2.22 |
|  | *XC_0433* | *ameR* | transcriptional regulator tetR/acrR family | +2.11 |
|  | *XC_1006* | *salR* | transcriptional regulator lacI family | +2.19 |
|  | *XC_1696* |  | transcriptional regulator | +2.56 |
|  | *XC_1964* | *tex* | RNA-binding transcription accessory protein | +2.12 |
|  | *XC_2723* | *ArsR* | transcriptional regulator ArsR family | +2.38 |
|  | *XC_3428* | *vanR* | transcriptional regulator gntR family | +2.29 |
| Replication and DNA metabolism | *XC_0109* |  | ATP-dependent DNA ligase | -3.25 |
|  | *XC_1499* | *holA* | DNA polymerase III delta subunit | -2.16 |
|  | *XC_3597* |  | DNA-binding protein | -3.03 |
|  | *XC_1684* |  | excinuclease ABC subunit C homolog | +2.05 |
|  | *XC_2785* |  | helicase | +2.37 |
| Transport | *XC_0174* | *yehZ* | ABC transporter amino acid permease | -2.29 |
|  | *XC_0218* |  | MFS transporter | -2.37 |
|  | *XC_1138* | *tolQ* | TolQ protein | -2.11 |
|  | *XC_1140* | *tolA* | TolA protein | -2.28 |
|  | *XC_1141* | *tolB* | TolB protein | -2.24 |
|  | *XC_1165* | *fepA* | TonB-dependent receptor | -2.01 |
|  | *XC_2178* | *nasA* | nitrate transporter | -3.05 |
|  | *XC_2512* |  | TonB-dependent receptor | -2.52 |
|  | *XC_2546* |  | MFS transporter | -2.75 |
|  | *XC_2547* |  | ABC transporter ATP-binding protein | -2.25 |
|  | *XC_2708* | *pstS* | ABC transporter phosphate binding protein | -2.15 |
|  | *XC_2928* | *catA* | cation transport protein | -2.08 |
|  | *XC_3063* | *fyuA* | TonB-dependent receptor | -3.12 |
|  | *XC_4000* | *tptC* | ABC transporter ATP-binding protein | -4.37 |
|  | *XC_4257* | *nodT* | outer membrane efflux protein | -2.67 |
|  | *XC_0820* | *dctA* | C4-dicarboxylate transport protein | +2.21 |
|  | *XC_1004* | *iroN* | TonB-dependent receptor | +4.73 |
|  | *XC_1104* | *iucA* | iron transporter | +3.67 |
|  | *XC_1644* | *btuB* | TonB-dependent receptor | +2.32 |
|  | *XC_2476* | *xylE* | MFS transporter | +4.62 |
|  | *XC_3458* | *nrtCD* | ABC transporter ATP-binding component | +4.07 |
|  | *XC_3459* | *nrtB* | permease | +2.09 |
|  | *XC_3463* | *phuR* | outer membrane hemin receptor | +2.37 |
|  | *XC_4146* | *ppa* | solute: Na^+^ symporter | +4.52 |
| Translation | *XC_0231* |  | acetyltransferase | -2.69 |
|  | *XC_1296* | *pip* | proline imino-peptidase | -2.03 |
|  | *XC_1422* |  | cysteine protease | -3.17 |
|  | *XC_1544* |  | metallopeptidase | -2.24 |
|  | *XC_2148* |  | metallopeptidase | -3.19 |
|  | *XC_2972* | *mucD* | periplasmic protease | -6.28 |
|  | *XC_3986* | *htrA* | protease Do | -2.23 |
|  | *XC_0534* | *groES* | 10kDa chaperonin | +2.87 |
|  | *XC_3550* |  | serine protease | +3.10 |
| Transcription | *XC_2281* | *fliA* | RNA polymerase sigma factor for flagellar operon FliA | +4.36 |
| Signal transduction | *XC_0113* |  | two-component system sensor protein | -2.53 |
|  | *XC_0114* |  | two-component system regulatory protein | -2.51 |
|  | *XC_0769* | *cpxA* | two-component system sensor protein, EnvZ family | -5.91 |
|  | *XC_0770* | *cpxR* | two-component system regulatory protein,OmpR family, | -4.61 |
|  | *XC_1149* | *exsG* | two-component system sensor protein | -2.60 |
|  | *XC_1150* | *exsF* | two-component system regulatory protein | -3.71 |
|  | *XC_1262* |  | two-component system sensor histidine kinase-response regulator hybrid protein | -2.24 |
|  | *XC_1421* | *creC* | two-component system sensor protein | -2.22 |
|  | *XC_3055* |  | two-component system regulatory protein | -2.31 |
|  | *XC_3056* |  | two-component system sensor protein | -2.24 |
|  | *XC_1261* |  | histidine kinase/response regulator hybrid protein | +2.59 |
|  | *XC_1938* | *gacA* | two-component system regulatory protein, NarL subfamily | +2.33 |
| Mobile genetic elements | *XC_3804* | *ISxac3* | ISxac3 transposase | -5.38 |
|  | *XC_0681* | *ISxac3* | ISxac3 transposase | +3.33 |
|  | *XC_1643* | *IS1479* | IS1479 transposase | +2.65 |
|  | *XC_2108* | *gV* | single-stranded DNA binding protein | +6.35 |
|  | *XC_2121* | *gVII* | minor coat protein | +26.18 |
|  | *XC_2124* | *gII* | phage-related protein | +7.16 |
| Pathogenicity and adaption | *XC_0008* | *tonB* | TonB protein | -2.06 |
|  | *XC_0052* | *avrBs2* | avirulence protein | -2.70 |
|  | *XC_0123* | *iroN* | TonB-dependent receptor | -2.05 |
|  | *XC_0672* |  | multidrug resistance efflux pump | -2.97 |
|  | *XC_0705* | *pgl* | endo-polygalacturonase | -2.12 |
|  | *XC_0714* | *prc* | tail-specific protease | -2.08 |
|  | *XC_1447* |  | extracellular serine protease | -5.56 |
|  | *XC_1450* |  | extracellular serine protease | -3.40 |
|  | *XC_1823* | *htpX* | heat shock protein | -2.17 |
|  | *XC_1849* | *pglA* | polygalacturonase | -2.64 |
|  | *XC_2004* | *avrXccC* | avirulence protein | -8.31 |
|  | *XC_2173* |  | exopolysaccharide biosynthesis protein | -4.50 |
|  | *XC_2324* | *pdeA* | c-di-GMP phosphodiesterase A | -4.33 |
|  | *XC_2506* | *csrA* | carbon storage regulator | -2.21 |
|  | *XC_2827* | *hpaR* | transcriptional regulator marR family | -2.25 |
|  | *XC_2837* | *pmrB* | multidrug resistance membrane translocase | -4.07 |
|  | *XC_3003* | *hrcC* | HrcC protein | -3.65 |
|  | *XC_3004* | *hrcT* | HrpB8 protein | -4.01 |
|  | *XC_3006* | *hrcN* | HrpB6 protein | -3.52 |
|  | *XC_3007* | *hrpB5* | HrpB5 protein | -4.44 |
|  | *XC_3009* | *hrcJ* | HrcJ protein | -4.16 |
|  | *XC_3010* | *hrpB2* | HrpB2 protein | -4.33 |
|  | *XC_3012* | *hrcU* | HrcU protein | -4.26 |
|  | *XC_3013* | *hrcV* | HrcV | -4.12 |
|  | *XC_3015* | *hrcQ* | HrcQ protein | -4.22 |
|  | *XC_3016* | *hrcR* | HrcR protein | -5.79 |
|  | *XC_3018* | *hpaA* | HpaA protein | -4.35 |
|  | *XC_3019* | *hrpD5* | HrpD5 protein | -3.74 |
|  | *XC_3022* | *hpaB* | HpaB protein | -3.52 |
|  | *XC_3023* | *hrpW* | HrpW protein | -3.90 |
|  | *XC_3025* | *hrpF* | HrpF protein | -3.54 |
|  | *XC_3076* | *hrpX* | HrpX protein | -4.66 |
|  | *XC_3657* | *copB* | copper resistance protein B precursor | -2.58 |
|  | *XC_3860* | *acrD* | acriflavin resistance protein | -2.84 |
|  | *XC_3861* | *acrA* | acriflavin resistance protein | -2.77 |
|  | *XC_3999* | *acrE* | acriflavin resistance protein | -3.34 |
|  | *XC_4223* | *OmpA* | OmpA-related protein | -2.89 |
|  | *XC_0104* | *aldA* | chloroacetaldehyde dehydrogenase | +2.13 |
|  | *XC_1005* |  | 1,4-beta-cellobiosidase | +4.90 |
|  | *XC_1027* | *virB6* | VirB6 protein | +2.93 |
|  | *XC_1119* | *bla* | beta lactamase | +2.06 |
|  | *XC_1298* | *pel1* | pectate lyase II | +2.29 |
|  | *XC_1432* | *mexA* | multidrug resistance protein | +3.37 |
|  | *XC_1789* | *gstA* | glutathione S-transferase | +2.74 |
|  | *XC_2798* | *mexF* | RND multidrug efflux transporter MexF | +3.04 |
|  | *XC_2799* | *mexE* | RND multidrug efflux membrane fusion protein | +3.90 |
|  | *XC_3590* | *pel2* | pectate lyase | +16.1 |
|  | *XC_3591* | *pel2* | pectate lyase | +14.61 |
|  | *XC_4200* |  | bleomycin resistance protein | +2.05 |
| Undefined category | *XC_2170* |  | putative epimerase | -2.98 |
|  | *XC_2413* |  | putative NTPase VagA | -2.34 |
|  | *XC_3158* |  | hydroxylase large subunit | -3.32 |
|  | *XC_3895* |  | putative disulphide-isomerase precursor | -3.24 |
|  | *XC_4294* |  | acetyltransferase | -3.63 |
| Conserved hypothetical protein | *XC_0034* |  | conserved hypothetical protein | -2.33 |
|  | *XC_0108* |  | hypothetical protein | -2.23 |
|  | *XC_0112* |  | conserved hypothetical protein | -5.33 |
|  | *XC_0176* |  | hypothetical protein | -3.51 |
|  | *XC_0217* |  | conserved hypothetical protein | -2.73 |
|  | *XC_0799* |  | conserved hypothetical protein | -4.70 |
|  | *XC_1077* |  | conserved hypothetical protein | -3.27 |
|  | *XC_1417* |  | conserved hypothetical protein | -2.04 |
|  | *XC_1713* |  | conserved hypothetical protein | -2.03 |
|  | *XC_1765* |  | conserved hypothetical protein | -2.09 |
|  | *XC_1852* |  | conserved hypothetical protein | -2.27 |
|  | *XC_2409* |  | conserved hypothetical protein | -2.13 |
|  | *XC_2587* |  | conserved hypothetical protein | -4.01 |
|  | *XC_2814* |  | conserved hypothetical protein | -3.34 |
|  | *XC_3128* |  | conserved hypothetical protein | -2.57 |
|  | *XC_3171* |  | conserved hypothetical protein | -2.28 |
|  | *XC_3173* |  | conserved hypothetical protein | -2.19 |
|  | *XC_3305* |  | hypothetical protein | -2.98 |
|  | *XC_0735* |  | conserved hypothetical protein | +2.53 |
|  | *XC_0792* |  | conserved hypothetical protein | +2.18 |
|  | *XC_0793* |  | conserved hypothetical protein | +3.26 |
|  | *XC_1023* |  | conserved hypothetical protein | +3.18 |
|  | *XC_1107* |  | conserved hypothetical protein | +2.21 |
|  | *XC_1188* |  | conserved hypothetical protein | +2.40 |
|  | *XC_1215* |  | conserved hypothetical protein | +2.33 |
|  | *XC_1220* |  | conserved hypothetical protein | +2.50 |
|  | *XC_1559* |  | conserved hypothetical protein | +2.08 |
|  | *XC_1697* |  | conserved hypothetical protein | +2.04 |
|  | *XC_1920* |  | conserved hypothetical protein | +2.26 |
|  | *XC_2123* |  | hypothetical protein | +10.16 |
|  | *XC_2230* |  | conserved hypothetical protein | +4.74 |
|  | *XC_2317* |  | conserved hypothetical protein | +2.08 |
|  | *XC_3149* |  | conserved hypothetical protein | +2.02 |
|  | *XC_3418* |  | conserved hypothetical protein | +2.18 |
|  | *XC_3970* |  | conserved hypothetical protein | +2.04 |

Note: False discovery rate (FDR) =0.05 and absolute value of log2FC(log2foldchange) =1 (equivalent to a fold change of 2) were used as the cut off values.“+” represents gene up-regulated in the ∆flp mutant, and “-”represents gene down-regulated.
