# Supplementary material for: Flp, a Fis‐like protein, contributes to the regulation of type III secretion and virulence processes in the phytopathogen Xanthomonas campestris pv. campestris
Source: Mol Plant Pathol. 2019 May 14;20(8):1119–33. doi: 10.1111/mpp.12818 (PMC6640185; doi:10.1111/mpp.12818)
Supplement: Supplementary file 4 — Table S3 Primers used in this study. [file MPP-20-1119-s004.docx]

**Table S3.** Primers used in this study^§^

| Primers | Nucleotide sequence (5′→3′) | The amplified fragment or the utilization |
| --- | --- | --- |
| L*flp*-F  L*flp*-R | ACAGTTGAATTCTGCAGCTGTCGCTGTCCGATGCCGA  ACAGTTGGATCCTGCGTTCAAAGGAGGTTCCCAGTGT | 333-bp DNA sequence upstream of *flp*, used for construction of *flp* deletion mutant. |
| R*flp*-F  R*flp*-R | ACAGTTGGATCCTATGGGCTGACGTAGGTTTGGGAATCGGG  ACAGTTAAGCTTTCAGGCGACGGTTGGGACGCGCTCG | 339-bp DNA sequence downstream of *flp*, used for construction of *flp* deletion mutant. |
| C*flp*-F  C*flp*-R | ACAGTTGGATCCGCGTGCTGCAGATCCAGGCCAGCTTCCGC  ACAGTTAAGCTTGGCACATGCGAGCGCCTTGCAGTCGATGG | DNA fragment of 297-bp *flp* coding sequence, used for complementation, overproduction and site-directed mutagenesis of *flp*. |
| Hflag-F  Hflag-R | GGGGGATCCTTGAACGCAGCCCCCTCTCGT  GGGAAGCTTCTACGTCAGCCCATACTCTTTATCGTCGTCATCTTTGTAGTCGATATCATGATCTTTATAATCACCGTCATGGTCTTTGTAGTC | DNA fragment encoding Flp fused with 3×Flag-tag N-terminally, used for constructing *Xcc* strain producing 3×Flag::Flp protein. |
| hrpXivt-F  hrpXivt-R | CGCATCGTCTGGCCTTCGTC  TTGCTGGAGGTGCTGCAGACCCTGT | 311-bp DNA fragment of *hrpX* spanning nucleotides from -131 to +179 relative to TIS, used for in vitro transcription assay. |
| 3076chipF  3076chipR | CGCATCGTCTGGCCTTCGTC  CGAAAGGATCATGCCGGGCT | 202-bp DNA fragment of *hrpX* promoter spanning nucleotides -131 to +70 relative to TIS, used for ChIP-PCR assay. |
| 0784F  0784R | CCAAGTAACCGCGGCGATCA  TCGCGCTCAGCACCAGATGT | DNA fragment of *XC_0784* promoter, used for ChIP-PCR assay. |
| 3076E-F  3076E-R | CGCATCGTCTGGCCTTCGTC  FAM-CGAAAGGATCATGCCGGGCT | 5’-FAM labelled DNA fragment of *hrpX* promoter spanning nucleotides from -131 to +70 relative to TIS, used for EMSA. |
| 3001-F  3001-R | GACGCGATCCATCGCAACAA  CGCGGGCGTTTCGGAATGAT | 219-bp DNA fragment spans nucleotides from 133 to 351 bp of the *XC_3001*, used for RT-PCR. |
| 2324-F  2324-R | TCAGTGACCAGACCGAAGTC  GTGCAGATCAGCGTCATCAC | 274-bp DNA fragment spans nucleotides from 194 to 467 bp of the *XC_2324*, used for RT-PCR. |
| 3657-F  3657-R | CATTGCTGGTGGCTCAATAGCTT  ACACTGCCGATACACCTTGACTT | 274-bp DNA fragment spans nucleotides from 255 to 528 bp of the *XC_3657*, used for RT-PCR. |
| 3129-F  3129-R | GCAGCCTGTTCTTCGCCCT  TGCGCAGCATGTCCGCGTC | 249-bp DNA fragment spans nucleotides from 101 to 349 bp of the *XC_3129,* used for RT-PCR. |
| 3597-F  3597-R | CAAGAATGCAAAGAAGCAGC  GCGGTATTTCGGTGCAACCT | 214-bp DNA fragment spans nucleotides from 51 to 264 of the *XC_3597*, used for RT-PCR. |
| 3437-F  3437-R | CAACTACATCGCCGAGGGTAA  TGTCGACGTCGATTTCGGGAG | 260-bp DNA fragment spans nucleotides from 303 to 562 bp of the *XC_3437*, used for RT-PCR. |
| 2004-F  2004-R | TTTGAGGCGGCCATATCACTCAGTG TCCTGAAATCGCTCCACACTGCCGA | 133-bp DNA fragment spans nucleotides from 411 to 543 bp of the *XC_2004*, used for RT-PCR. |
| 3694-F  3694-R | AGCGCCAGCTACTACATCAAT  ACTCGTAGTAGCCCACGCCCA | 199-bp DNA fragment spans nucleotides from 235 to 433 bp of the *XC_3694*, used for RT-PCR. |
| 2827-F  2827-R | TTGATCCAACCGCTAAACGC  ACTTCGGTTGGCGTGATTGC | 217-bp DNA fragment spans nucleotides from 11 to 227 bp of the *XC_2827*, used for RT -PCR. |
| 0158-F  0158-R | GCAAGCGCTACTACATCTAT  CATGTAGTACCAGCCGTTGT | 238-bp DNA fragment spans nucleotides from 341 to 678 bp of the *XC_0158*, used for RT-PCR. |
| 2659-F  2659-R | TGTGCCGTACGCGCGTGATC  CGGTGCACCATTCCATTCGGG | 317-bp DNA fragment spans nucleotides from 911 to 1227 bp of the *XC_2659*, used for RT-PCR. |
| 1273-F  1273-R | CGTTTCCGTATTACTACATGAC  AGCCGATCAGGATGGTGTGC | 231-bp DNA fragment spans nucleotides from 152 to 382 bp of the *XC_1273*, used for RT -PCR. |
| 1314-F  1314-R | TGCTGCGCGACTTCGAGTTGA  ACATCACCGCGCAGGCGCATT | 220-bp DNA fragment spans nucleotides from 131 to 350 bp of the *XC_1314*, used for RT-PCR. |
| 3652-F  3652-R | ACTGCCTTCAAGATCCGCGG  TTGAAGCCGGTGGACAACGC | 194-bp DNA fragment spans nucleotides from 436 to 629 bp of the *XC_3652*, used for RT-PCR. |
| 0783-F  0783-R | GCAACCACTACGCGTGGGTG  TAATGCCAGCCGCGCACGAT | 154-bp DNA fragment spans nucleotides from 152 to 305 bp of the *XC_0783*, used for RT-PCR. |
| 3003-F  3003-R | CCGTGGCACTCGCGCAGCT  CGCCCAAACTCAAGGTCGC | 262-bp DNA fragment spans nucleotides from 112 to 373 bp of the *XC_3003* (*hrpA*), used for RT-PCR. |
| 3011-F  3011-R | GCGAGTACTGCGGCCAGAGTTGAAG  AAACAGGCAACACGCGTACAAGGCC | 160-bp DNA fragment spans nucleotides from 111 to 270 bp of the *XC_3011 (hrpB)*, used for RT -PCR. |
| 3012-F  3012-R | GGCAGCTATTTTGTCGAACATC  CTGCACAAAGGTGCCGATCAAT | 183-bp DNA fragment spans nucleotides from 136 to 318 bp of the *XC_3012* (*hrpC*), used for RT-PCR. |
| 3015-F  3015-R | GGCGCTGCTGTTGAACGA  ATATCGAGGCGATCGACC | 156-bp DNA fragment spans nucleotides from 141 to 296 bp of the *XC_3015* (*hrpD*), used for RT-PCR. |
| 3021-F  3021-R | AGTCTGATGGGTGGCATTGGTAAAT  GTTGAGCGCCACGTTGAAGTCAAGT | 102-bp DNA fragment spans nucleotides 121 to 222 bp of the *XC_3021* (*hrpE*), used for RT-PCR. |
| 3025-F  3025-R | AGCCGATTGAGAAACGGACCTCCT  GCGGCGTCTTGTCTTTGTGCTGATT | 183 -bp DNA fragment spans nucleotides from 257 to 439 bp of the *XC_3025* (*hrpF*),used for RT–PCR. |
| 3076F  3076R | CTGGAACGCGCGCAAGAGGTGGTTT  CTCCACCAGCAATGCGCAGCAGATT | 286 -bp DNA fragment spans nucleotides from 436 to 747 bp of the *XC_3076* (*hrpX*), used for RT–PCR |
| 3077F  3077R | CACGAAGTGCTCAGCCTGCCAGTCA  ACTCGGTATGGCTACCCCAGACTGC | 286 -bp DNA fragment spans nucleotides from 436 to 747 bp of the *XC_3077* (*hrpG*),used for RT–PCR |
| 16SF  16SR | GCCTAACACATGCAAGTCGAACGGC  AATATTCCCCACTGCTGCCTCCCG | 325-bp DNA fragment of the 16S rDNA sequence, used for RT-PCR. |

§The underlined sequences indicate the restriction sites for *Bam*HI, *Eco*RI, *Hin*dIII, and *Xba*I, respectively.
